# Cold‐active microbial enzymes and their biotechnological applications

**DOI:** 10.1111/1751-7915.14467

**Published:** 2024-04-24

**Authors:** Mohammed Kuddus, Roohi, Naushin Bano, Gouse Basha Sheik, Babu Joseph, Burhan Hamid, Raveendran Sindhu, Aravind Madhavan

**Affiliations:** ^1^ Department of Biochemistry, College of Medicine University of Hail Hail Saudi Arabia; ^2^ Protein Research Laboratory, Department of Bioengineering Integral University Lucknow India; ^3^ Department of Microbiology Gesco Healthcare Pvt. Ltd. Chennai India; ^4^ Department of Clinical Laboratory Sciences, College of Applied Medical Sciences Shaqra University Shaqra Saudi Arabia; ^5^ Center of Research for Development University of Kashmir Srinagar India; ^6^ Department of Food Technology TKM Institute of Technology Kollam Kerala India; ^7^ School of Biotechnology Amrita Vishwa Vidyapeetham, Amritapuri Kollam Kerala India

## Abstract

Microorganisms known as psychrophiles/psychrotrophs, which survive in cold climates, constitute majority of the biosphere on Earth. Their capability to produce cold‐active enzymes along with other distinguishing characteristics allows them to survive in the cold environments. Due to the relative ease of large‐scale production compared to enzymes from plants and animals, commercial uses of microbial enzyme are alluring. The ocean depths, polar, and alpine regions, which make up over 85% of the planet, are inhabited to cold ecosystems. Microbes living in these regions are important for their metabolic contribution to the ecosphere as well as for their enzymes, which may have potential industrial applications. Cold‐adapted microorganisms are a possible source of cold‐active enzymes that have high catalytic efficacy at low and moderate temperatures at which homologous mesophilic enzymes are not active. Cold‐active enzymes can be used in a variety of biotechnological processes, including food processing, additives in the detergent and food industries, textile industry, waste‐water treatment, biopulping, environmental bioremediation in cold climates, biotransformation, and molecular biology applications with great potential for energy savings. Genetically manipulated strains that are suitable for producing a particular cold‐active enzyme would be crucial in a variety of industrial and biotechnological applications. The potential advantage of cold‐adapted enzymes will probably lead to a greater annual market than for thermo‐stable enzymes in the near future. This review includes latest updates on various microbial source of cold‐active enzymes and their biotechnological applications.

## Graphical abstract

Microorganisms known as psychrophiles/psychrotrophs, which survive in cold climates, constitute majority of the biosphere on Earth. Microbes living in these regions are important for their metabolic contribution to the ecosphere as well as for their enzymes, which may have potential industrial and biotechnological applications. This review includes latest updates on various microbial source of cold‐active enzymes and their biotechnological applications.

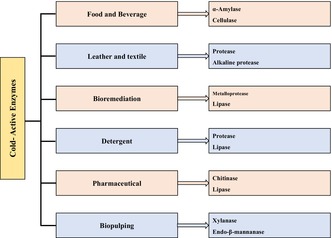

## INTRODUCTION

Most of the ecosystems on the Earth are subjected to temperatures that are consistently below 5°C. The ecosphere's cold ecosystems, which include seas, soils, glaciers, lakes, and sea ice, make up more than 70% of the total area (Feller & Gerday, 2003). The vast majority of Earth's biosphere is made up of microbes called psychrophiles and psychrotrophs that can survive in extremely cold conditions. True psychrophiles are adapted not only to low temperatures but also, sometimes, to additional environmental limitations. They are subjected to extraordinarily high pressures in the ocean depths and sediments; hence, they must be called as piezo‐psychrophiles as well (Yayanos, 1995). Microorganisms that have adapted to the cold are a possible source of heat‐sensitive, homologous cold‐active enzymes that have high catalytic effectiveness at low and mild temperatures. The potentials of cold‐active enzymes and their producing organisms have been periodically evaluated, and they have a wide range of current and future applications in biotechnology (Kuddus, 2015). These enzymes are highly catalytic at low and moderate temperatures where homologous mesophilic enzymes are inactive. In biotechnology, this characteristic shortens low‐temperature process durations, saves energy, prevents chemical transformations, and minimizes volatile molecular loss. Cold‐active enzymes support low‐temperature processes that preserve heat‐labile compounds and maintain product quality. It allows selective enzyme inactivation in complicated media without expensive heating/cooling equipment (Margesin, 2009).

**FIGURE 1:**
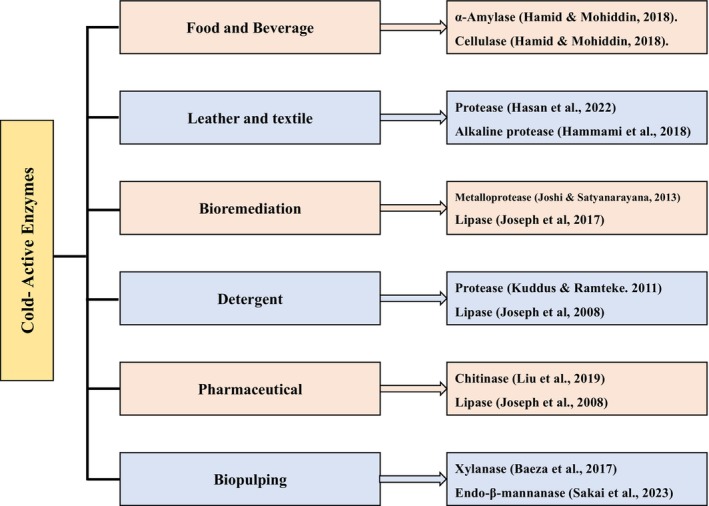
Cold‐active enzymes and their biotechnological applications.

Scientific and industrial groups have put in a lot of work over the past few decades to find new cold‐active enzymes with desirable features for usage in various biotechnological procedures (Mangiagalli & Lotti, 2021). Molecular biology, detergents, food and drink processing can all benefit from the cold‐adapted enzymes because of their increased activity at lower temperatures. In addition, the selective deactivation of enzymes in complicated combinations is possible through the use of thermo‐sensible enzymes. The ability to perform biotechnological activities more efficiently, economically with sustainability than with high‐temperature‐adapted enzymes is what makes them so appealing and in demand (Barroca et al., 2017). The food, beverages, biofuels, and detergent sectors all make use of distinct techniques that call for psychrozymes such as amylases, cellulases, invertases, proteases, and lipases. In addition to its use in lactose‐free dairy products, cold‐active galactosidases have found a home in the culinary, cosmetic, and pharmaceutical industries. These enzymes are distinguished by their optimum catalytic activity at temperatures between those considered low and adequate; nevertheless, they are also heat‐sensitive and rapidly inactivated over a certain temperature (Hamid et al., 2022). Due to their high efficiency and environmentally friendly nature, enzymes are in great demand in many industrial applications, including food and beverage production (Grzonka et al., 2007; Moharram et al., 2022; Schäfer et al., 2006; Singh et al., 2016). Moreover, these enzymes are an essential component of a well‐established global sector that is projected to increase in value over the next several years to US$6.3 billion (Dewan, 2017; Moharram et al., 2022; Singh et al., 2016). These days, it's common practice to use cold‐active enzymes to bring down the temperature of industrial processes. This reduces energy consumption and carbon emissions, and it also increases productivity because these processes work better at ambient or lower temperatures (Białkowska & Turkiewicz, 2014; Pulicherla et al., 2011; Sarmiento et al., 2015). Cold‐active enzymes are employed in biotechnology to limit the loss of volatile components and stop a variety of undesirable reactions (Gerday, 2013; Kuddus, 2018; Kumari et al., 2021; Santiago et al., 2016). It is anticipated that the application of cold‐active enzymes would significantly grow during the coming decades (Al‐Ghanayem & Joseph, 2020; Santiago et al., 2016). Cold‐active enzymes with different biotechnological applications and examples are shown in Figure 1.

## HABITATS OF COLD‐ADAPTED MICROORGANISMS AND THEIR COLD‐ACTIVE ENZYMES

All the cold‐adapted microorganisms grouped into two broad categories viz. psychrophiles, which thrive at temperatures below 15°C and psychrotrophs, which can survive at temperatures between 20 and 25°C (Farrell & Rose, 1967; Moyer & Morita, 2007). The name “psychrophile” is of Greek origin (“Psychros” means cold and “Philos” means loving), i.e. cold‐loving (Schmidt‐Nielsen, 1902). Psychrophiles are usually found in deep oceans, icebergs, and frozen areas of land such as glaciers and snow‐covered areas; and also isolated from cold‐water lakes, cold deserts, and caves where the temperature is below 10°C (Hamid et al., 2022). About 85% of Earth is covered by various cold habitats, both manmade and natural, and within these environments, a wide variety of cold‐adapted microorganisms including archaea, bacteria, fungi, yeasts, and viruses are thriving (Margesin & Collins, 2019). Almost three‐quarters of all known forms of life on the Earth are psychrophiles, which survive in extreme environments such as Antarctica, high‐altitude soils, glaciers, deep sea waters (both fresh and marine), and the mountains (Hamid et al., 2022).

Most of the cold‐active enzymes are isolated from cold‐adapted microorganisms; however, some enzymes active at low temperatures were isolated from mesophilic microorganisms (Bhatia et al., 2021; Hamid et al., 2022; Santiago et al., 2016). These enzymes have high catalytic efficiency and protein flexibility at low temperature along with low thermal stability due to their unique protein characteristics (Rojas‐Contreras et al., 2015). On the other hand, some of the cold‐active enzymes showed high thermal stability that have an added advantage for various industrial applications (Zhou et al., 2019). Microorganisms such as bacteria, fungi, and algae were found to be excellent sources of cold‐active enzymes and their sources have been reviewed (Bhatia et al., 2021; Hamid et al., 2022; Santiago et al., 2016). A list of cold‐active enzymes recently isolated from various cold‐adapted microorganisms and their habitat is summarized in Table 1. Five psychrophilic Gram‐negative bacteria were isolated from maritime sediments of Svalbard by Knoblauch et al. (1999). Sánchez et al. (2009) isolated thirty bacterial colonies from South Atlantic Argentina. A promising source of antibacterial activity has been discovered in the isolated bacterium, closely related to *Serratia* sp. (Sánchez et al., 2009). Metabolically active psychrophiles capable of reproducing at −15°C (*Planococcus halocryophilus* Or1) were isolated from Arctic permafrost (Mykytczuk et al., 2013). The above‐mentioned microorganisms are considered unmined treasures for industrial and biotechnological applications. Unculturable cold‐adapted microbes that have various potentials in producing metabolites are explored through the metagenomics approach (Vester et al., 2015). This helps to increase knowledge and gives us a better understanding of novel enzymes that can be used in biotechnological applications.

**TABLE 1:** Some important cold‐active enzymes and their microbial source.

Cold‐active enzyme	Cold‐adapted microorganism	Habitat of microorganism	Temp. of habitat	Reference
α‐amylase	*Microbacterium foliorum* and *Bacillus cereus*	Gangotri glacier, India	2–5°C	Kuddus et al. (2012)
β‐galactosidase	*Alteromonas* sp. ML117	Deep sea Mariana Trench	NM	Yao et al. (2019)
β‐d‐galactosidase	*Arthrobacter* sp. 32c	Antarctica	2–4°C	Rutkiewicz et al. (2019) Hildebrandt et al. (2009)
*β*‐galactosidases (4 different types)	*Cryobacterium* sp. LW097	Subglacial sediments northwest of China	≤5°C	
β‐Galactosidase	*Rahnella inusitata*	Antarctica	2–4°C	Núñez‐Montero et al. (2021)
Carboxymethyl Cellulase	*Rhodotorula mucilaginosa* BPT1	Baramullah (J&K) India	≤5.0°C	Parmar et al. (2023)
Cellulase	*Exiguobacterium sibiricum* K1	Agro‐residual waste	NM	Kumari et al. (2023)
Cellulase	*Myxobacterial Strain*	Mirgund Wetland from the North‐Western Himalayas	2–5°C	Dhanjal et al. (2023)
Chitosanase	*Pseudoalteromonas* sp. SY39	Marine environment	NM	Zhou et al. (2019)
Chitinase	*Trichoderma gamsii R1*	Shrimp shell waste	NM	Wang et al. (2023)
Glycosyltransferase WapH	*Pseudomonas extremaustralis*	Antarctica	2–4°C	Benforte et al. (2018) Tribelli et al. (2020)
Haloalkane dehalogenase DpcA	*Psychrobacter cryohalolentis* K5	Cryopeg within permafrost; Kolyma lowland Siberia	0°C	Tratsiak et al. (2019) Bakermans et al. (2006)
Laccase	*Psychrobacter* sp. NJ228	Antarctica	2–4°C	Zhang et al. (2023)
Lipase	*Pseudoalteromonas* sp.	Antarctic sea ice	2–3°C	Wang et al. (2012)
Lipase	*Pseudomonas marinensis*	Deep‐sea sedimental samples, China	NM	Guo et al. (2021)
Lipase	*Bacillus sphaericus* MTCC 7526	Himalayan glacier soil, India	2–5°C	Joseph and Ramteke (2013)
Lipase	*Microbacterium luteolum*	Himalayan glacier soil, India	2–5°C	Joseph et al. (2012)
Lipase	*Microbacterium phyllosphaerae*	Himalayan glacier soil, India	2–5°C	Joseph et al. (2008)
Lipase	*Pseudomonas* sp. A6	Water samples Mediterranean Sea in Alexandria, Egypt	22°C	Abdella et al. (2023)
Lipase Protease	*Psychrobacter* sp. 94‐6PB	Antarctic glacier	−10°C	Perfumo et al. (2020)
Lipase/esterase	*Halocynthiibacter arcticus*	Marine sediment of the Arctic	−2 to 8°C	Le et al. (2020)
Nitrous oxide reductase	*Arcobacter* and *Herminiimonas*	Arctic fjord sediments (Svalbard, Norway)	1.3°C	Canion et al. (2013)
Oligoribonuclease	*Colwellia psychrerythraea* 34H	Arctic marine sediments	−2 to 8°C	Lee, Park, et al. (2019) and Lee, Son, and Kim (2019)
Oxygenases	*Pseudomonas* S2TR‐14	Contaminated cold‐climate site	NM	Miri et al. (2021)
Pectinase	*Cladosporium parasphaerospermum* *C*. *chlamydosporigenum*, *C*. *compactisporum*	Sohag city, Egypt	10°C	Moharram et al. (2022)
Polygalacturonase	*Thalassospira frigidphilosprofundus* S3BA12	Depth of 1000 m in the Bay of Bengal, Chennai, India	≤5.0°C	Adapa et al. (2019)
Protease	*Chryseobacterium polytrichastri*	East Rathong Glacier Sikkim India	≤5°C	Mukhia et al. (2021)
Protease	*Marinobacter psychrophilus*	Canadian Basin	1–2°C	Zhang, Li, Xin, Chi, et al. (2008), Zhang, Li, Xin, Liu, et al. (2008), and Zhang, Liu, Xin, Yu, et al. (2008)
Protease	*Stenotrophomonas maltophilia*	Gangotri glacier (Western Himalaya)	2–5°C	Kuddus and Ramteke (2009)
Protease	*Curtobacterium lutium*	Gangotri glacier (Western Himalaya)	2–5°C	Kuddus and Ramteke (2008)
Protease	*Clostridium schirmacherense*	Lake sediment of Schirmacher Oasis, Antarctica	2–4°C	Alam et al. (2006)
Protease	*Bacillus pumilus*	Himalayan glacier soil, India	2–5°C	Farooq et al. (2021)
Protease (Pro21717)	*Pseudoalteromonas arctica* PAMC 21717	Polar and Alpine Microbial Collection (PAMC)	NM	Park et al. (2018)
Protease	*Chryseobacterium soli*	Samples from Cold storages	2–5°C	Mageswari et al. (2017)
RNase R	*Psychrobacter* sp. ANT206	Antarctic sea‐ice (68° 300 E, 65° 000 S)	2–3°C	Wang et al. (2019)
Transglutaminase	*Penicillium chrysogenum*	Antarctica	2–4°C	Glodowsky et al. (2020)
Keratinase	*Arthrobacter oryzae* BIM B‐1663	Antarctic green snow	NM	Smirnova et al. (2023)

Abbreviation: NM, not mentioned.

## COLD‐ACTIVE ENZYMES IN FOOD PROCESSING/FOOD INDUSTRY

**FIGURE 2:**
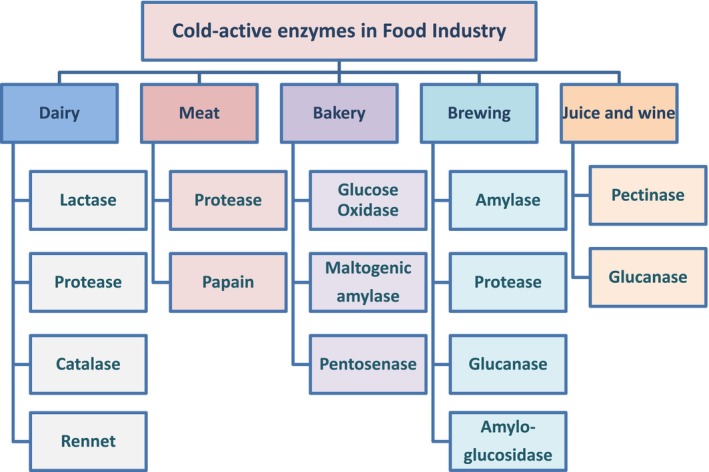
Cold‐active enzymes used in different sectors of food industry.

For the food scientists and biotechnologists, the current issue is the discovery of novel enzymes for their commercial applications. Cold‐active enzymes are excellent biocatalysts with strong specific activity at low temperature that do not require heating processes, which impede the quality, sustainability, and cost‐effectiveness of industrial production. The cold‐shock proteins of psychrophilic bacteria, which include enzymes like pectinase, proteases, amylases, lipases, and cellulases, have numerous biotechnological applications in the food‐processing sector (Gounot, 1991). Cold‐active galactosidase with an optimal temperature range of 15–18°C has opened a new field of research in the dairy and food processing industry (Hamid et al., 2022). Some of the important cold‐active enzymes used in the food industry are described below and summarized in Figure 2.

### Lactase

Lactose, a type of carbohydrate, makes up the bulk of our daily diet. β‐galactosidase, more generally known as lactase, is an enzyme responsible for hydrolysing lactose into glucose and galactose (Shukla & Wierzbicki, 1975). The food processing industry is using β‐galactosidase at large scale. Cold‐adapted β‐galactosidase is a significant food enzyme, which removes lactose from the milk at low temperatures or in refrigerators so that it can be consumed by lactose‐intolerant peoples. Also, the cheese industry's waste product, whey, is converted to more easily fermentable glucose and galactose using cold‐adapted β‐galactosidase. Recent studies have characterized a marine psychrophilic bacterium, which produces cold‐active β‐galactosidase that digest over 80% of the lactose in raw milk at 20°C and pH 6.5, indicating the possibility of using them in the dairy sector at industrial scale (Ghosh et al., 2012; Pulicherla et al., 2013). Potential cold‐active β‐galactosidases could be used to produce low‐cost lactose‐free products.

### Amylase

Amylase is one of the most essential industrial enzymes used in food, textile, paper, and fermentation industries. In biotechnology, these enzymes are especially used for the hydrolysis of starch. Cold‐active amylases present novel possibilities for biotechnological exploitation due to their strong catalytic activity at low temperature, low thermostability, and peculiar specificities. *Alteromonas haloplanktis*, an Antarctic bacterium, was the first microorganism producing cold‐adapted α‐amylase, which was extensively studied and has been magnificently expressed in the mesophilic host *E*. *coli* (Feller et al., 1998). Kuddus et al., isolated cold‐active α‐amylase producing *Microbacterium foliorum* from Gangotri glacier, Western Himalaya, India with potential biotechnological applications (Kuddus et al., 2012). The structure and function of cold‐active α‐amylases and their biotechnological potentials are reviewed by Kuddus et al. (2011).

### Pectinase

The pectinase or pectinolytic enzymes are crucial components of the food processing industry. Pectinolytic enzymes, which are utilized in the food, paper, textile, and juice industries, make up around 10% of the enzyme market (Garg et al., 2016; Kashyap et al., 2001; Semenova et al., 2006). The juice company applies low temperatures (15°C) to reduce cloudiness and bitterness in fruit juices to conserve energy, retain labile and volatile taste components, and stop the growth of harmful microorganisms (Kashyap et al., 2001; Pulicherla et al., 2011). Because grape juice and other fruit juices must have a pH of between 2.5 and 3.5, researchers have been searching for pectinases that can function at both low temperatures and low pH (Adapa et al., 2014). Many studies have been done on the isolation and characterization of cold‐active pectinases from microorganisms. Recently, three new species producing cold‐active pectinases viz *Cladosporium parasphaerospermum*, *Cladosporium chlamydosporigenum*, and *Cladosporium compactisporum* are reported (Moharram et al., 2022). The purified cold‐active pectinase enhanced the yield of apple, orange, apricot, and peach juice and improved the clarity and colour of orange juice (Moharram et al., 2022). Another study reported pectinases from Antarctic *Geomyces* sp. F09‐T3‐2 that may be potentially suitable for biotechnological applications needing cold‐active pectinases (Poveda et al., 2018). Pectinases are also used to improve the cloud stability of fruit nectars, clarify fruit juices and wines, and process coffee and tea (Reid & Ricard, 2002; Soares, 2001).

### Lipase

For the esterification of fats and the generation of fatty acids, cold‐active lipases are used (Jaeger and Eggert, 2002). The lipases produced by psychrophilic bacteria are a crucial enzyme in today's food processing industries and become an integral part of the industry in many ways. Various cold‐active lipase‐producing microorganisms and their source are already reported (Kavitha, 2016). Cold‐active lipases have been utilized to change flavours and enhance the consistency of many foods. To generate antioxidants for use in sunflower oil, functionalized phenols were esterified using cold‐active lipases from *C*. *antarctica*, *Candida cylindracea* AY30, *Hansinuela lanuginosa*, *Pseudomonas* sp., and *G*. *candidum* (Buisman et al., 1998; Pandey et al., 1999). Cold‐active lipase from *Pseudomonas* strain P38 is commonly employed in nonaqueous biotransformation for butyl caprylate n‐heptane production (Tan et al., 1996).

### Protease

The most important use of cold‐active proteases, which are produced by psychrophilic microorganisms, is in developing the flavour and enhancing the tenderness of meats stored in the refrigerator. In the food industry, cold‐active proteases are useful due to their ability to maintain product integrity at low temperatures. Rapid inactivation of thermally labile enzymes, such as cold‐active proteases, can be achieved by subjecting them to mild heat (Margesin et al., 2002) which is beneficial in the process management. Protein hydrolysates that are both highly digestible and nutritionally beneficial can be prepared by using cold‐active alkaline proteases at low temperature. Protein hydrolysate is beneficial in blood pressure management and as an ingredient in infant food formulas and also used to fortify fruit juices and soft beverages for medicinal purposes (Neklyudov et al., 2000). Other possible applications of cold‐active proteases in the food industry are reviewed by Yang et al. (2023).

## COLD‐ACTIVE ENZYMES IN DETERGENT INDUSTRY

Household or industrial dirt and stains mainly in the form of proteins, lipids, and starch components are difficult to remove from the fabrics. Most of these stains are removed by heating and beating which shortens the life of fabrics (Rahman et al., 2023). Enzyme‐based detergents play an important role in the removal of dirt/stains. However, in the cold regions it is difficult to remove the dirt/stains by using detergents that contain mesophilic enzymes. The addition of chemicals to replace the enzymes increases environmental pollution and is harmful. Also, mesophilic enzymes with narrow physiochemical activities become inactive under low temperatures. In such conditions, cold‐active enzymes are gaining more attention due to their low‐temperature activity and thermostability (Hamid et al., 2022). Enzymes active under low temperatures with alkaline stability help to retain the quality of fabrics and fulfil consumer needs. Further cold‐active enzymes improve washing performance with low energy requirements and preserve the quality and life of fabrics. These enzymes are biodegradable, which reduce pollution and are safe for aquatic organisms. Washing machine manufacturers are targeting machines to perform cold washing and thereby reducing energy consumption and protecting the fabrics (Sarmiento et al., 2015).

Enzymes such as lipase, protease, and amylase are normally used as detergent additives. Lipases hydrolyse lipids and thereby it removes the lipid stains. Cold‐active lipases are capable of removing the lipid stain even at low temperatures. Proteases are enzymes that hydrolyse peptide bonds. The protein stains such as egg stains, bloodstains, grass, etc. are removed by the addition of cold‐active proteases in the detergent. Several cold‐adapted microorganisms isolated from different cold regions have been reported for cold‐active protease production (Kuddus & Ahmad, 2012; Kuddus & Ramteke, 2011). Some stains, such as sauces, vegetable stains, gravy, cereals, etc., are starch‐based stains and are removed easily by using amylases as a detergent additive (Niyonzima & More, 2014). Amylases break the bonds between glucose units in the linear amylose chain of starch by cleaving glycosidic linkages. Amylases contribute around 25% of total enzyme sales and are used in 90% of the detergent formulation (Hmidet et al., 2009). Some studies on detergent‐resistant pullulanase, which hydrolyse the α‐1,6 glucosidic linkages, are also reported (Wu et al., 2023). Along with cold‐active protease and lipase, cellulase is also used as a detergent additive that helps in removing stains by cleaving cellulose polymers. It increases the brightness of the fabric, along with reducing the fuzz and pills, especially in woollen garments. Cellulose is also used for increasing the finishing and smoothness of fabrics (Karmakar & Ray, 2011). In cotton fabrics, dirt and damaged fibres are accumulated in between cotton fibres. The dirt present in inter‐fibrils is also removed or digested by enzymatic action (Niyonzima, 2019). Mannanase and pectin stains are also difficult to remove from fabrics. Cold‐active mannanase and pectinase have a great importance to be an additive in detergents to improve the efficiency of detergent formulations (Kim et al., 2008). However, till date cold‐active pectinases and mannanase for its compatibility with detergents are not well explored.

## COLD‐ACTIVE ENZYMES IN TEXTILE INDUSTRY

In the textile industries, enzymes including cellulases, catalases, and laccases are frequently used. These enzymes are capable of destroying starch, degrading excessive hydrogen peroxide, decomposing materials, and tainting lignin. Cold‐active enzymes are increasingly being used in the textile industry due to their extraordinarily clear, productive, non‐toxic, and environment friendly qualities. Some of the cold‐active enzymes generally used in the textile industry include cellulases and proteases.

Cellulases catalyse the hydrolysis of cellulose into shorter oligosaccharides and ultimately in glucose. In the textile industry, cold‐active cellulase plays a critical role economically in the biopolishing of fabric and stone‐washing of jeans at low temperatures (Gerday et al., 2000). It is also used to remove fine fluff on the surface of cotton fabric under low‐temperature conditions (Bhat et al., 2013). Proteases are used in the textile industry to increase the softness of raw silk by removing its stiff and unappealing gum coating of sericin. The surface of wool and silk fibres can be altered by protease treatments to provide novel and distinctive finishes (Benmrad et al., 2018). Textile companies can make advantage of cold‐active proteases (Singhal et al., 2012). When every step is carried out at low temperature, cloth can be protected from the damaging effects of high temperatures for an extended period. Because synthetic fibres cannot withstand temperatures beyond 50–60°C, cold‐active proteases provide us with a novel and unique method of clearance of new fabrics (Guduk et al., 2019; Joshi & Satyanarayana, 2013).

## COLD‐ACTIVE ENZYMES IN WASTE‐WATER TREATMENT

Wastewater contains different types of chemicals, oils, polysaccharides, and other contaminants that reduce the oxygen content, which leads to the death of aquatic organisms (Bashir et al., 2020). A high content of these materials even affects the treatment of wastewater. Wastewater discharge from households, restaurants, and food industries in cold regions needs more attention due to the formation of clogs and flocs (Mahto & Das, 2022). Emulsified oil in the wastewater of cold regions has density variation and is more viscous with lesser interfacial films to stabilize the oil phase. To separate the flocs needs high‐cost treatment, in this context use of cold‐active enzymes in pretreatment reduces the issues at low temperatures. Wastewater treatment by using cold‐active enzymes is expensive due to the costly process of enzyme production. An alternative method such as inoculating cold‐adapted enzyme‐producing bacteria or cell immobilization or enzyme immobilization reduces the costs of treating the wastewater at low temperature.

Cold‐active lipases play an important role in reducing oil contamination in wastewater (Hassan et al., 2018). Lipases act on the interface between the substrate and aqueous phase and different types of lipases with unique substrate specificity are essential to dissolve lipid contaminants in the wastewater (Nimkande & Bafana, 2022). Another major component of wastewater is starch molecules that are hydrolysed into smaller glucose units with the help of alpha‐amylase enzymes. Chemical degradation of starch molecules is now substituted by microbial amylase due to its substrate specificity, stability, and easy manipulation. Cold‐active amylase with pH and substrate stability can be used in the treatment of wastewater in cold regions. Another major concern is proteinaceous waste from food and household effluents that interferes with BOD in the aquatic system. These materials can be treated with crude enzymes or direct microbial inoculation of cold‐adapted proteolytic bacteria for reducing the production cost of pure cold‐active enzymes (Furhan, 2020). Wastewater from textile, animal feed and paper industries contains cellulose and related components. Cold‐active cellulase plays an important role in hydrolysing cellulose materials in wastewater. Biosurfactants are also used in the treatment of wastewater, that reduce the surface tension between the air‐water interface and bioemulsifiers that reduce the interfacial tension between immiscible liquids or solid materials. These biosurfactants and bioemulsifiers increase the availability of substrates to the enzymes even in cold climatic conditions. Cold‐active enzymes can be used in the treatment of waste materials from candy, confectionary, leather, paper, meat and wine industries in cold regions (Duarte et al., 2018). Cold‐active pectinase (polygalacturonase) was produced from *Geotrichum* sp by using waste from the fruit and vegetable industries (Divya & Padma, 2015). Cold‐tolerant chitinases were produced from Antarctic fungus *Lecanicillium muscarium* CCFEE‐5003 by using shrimp and crab wastes, so it may be used in the treatment of chitin‐containing wastewater from shrimp and crab industries (Barghini et al., 2013). Apart from these applications, cold‐active enzymes are also reported for treating harmful chemicals such as petroleum hydrocarbons, alkanes, aromatic hydrocarbon, chlorinated hydrocarbons, polycyclic aromatic hydrocarbons, and petroleum hydrocarbons in wastewater in cold regions (Park & Park, 2018).

## COLD‐ACTIVE ENZYMES IN BIOPULPING

Biopulping is an environment friendly process that employs biological methods in lieu of chemical agents to separate lignin from cellulose fibres within wood chips, with the aim of reducing use of chemicals and enhancing sustainability (Worku et al., 2023). It involves the microbial pretreatment of wood chips with the concept centred on the colonization of wood by microbes that selectively degrade lignin while largely preserving cellulose (Ferraz et al., 2008; Mendonça et al., 2008). Conventional pulping methods heavily rely on the utilization of toxic chemicals like sodium hydroxide (NaOH) and sodium sulfide (Na_2_S), which can result in adverse environmental consequences. In contrast, biopulping predominantly uses fungi, enzymes, and biological methods to break down lignin, promoting an environmentally friendly approach (Worku et al., 2023). However, in the context of biopulping, there are instances where controlled and limited chemical usage may persist. This could involve the application of enzymes or other biological agents that have been derived from chemical processes. Moreover, chemicals may occasionally play a role as facilitators in the biopulping process to enhance efficiency or accelerate reactions (Kumar et al., 2020). Biotreatment can yield several benefits, including reduced pulping time, lower consumption of bleaching chemicals, improved pulp strength, enhanced delignification rates, decreased alkali usage, and energy savings during defibration and refining phases (Islam et al., 2008; Mendonça et al., 2008). These advancements offer solutions to certain challenges in traditional chemical and mechanical pulping methods. Combining biopulping with mechanical pulping results in a sustainable approach that significantly increases mill throughput and reduces electrical energy consumption while producing stronger pulp with longer fibres and increased fibrillation. The introduction of specific lignin‐degrading microorganisms can rapidly modify wood cell walls (Bajpai, 2018).

Biopulping has the potential to revolutionize the paper and pulp industry by offering a more sustainable alternative to traditional pulping methods. The use of biological methods instead of chemicals can reduce the environmental impact of the pulping process and potentially lead to improved product quality (Bhardwaj et al., 2019). While biopulping is currently in its early stages of development, it holds significant promise as a more sustainable alternative to conventional pulping methods. The biopulping process with microbial cold enzymes typically entails introducing these enzymes to the wood chips, allowing them to react over a specified period. Subsequently, the fibres undergo a washing step to eliminate any remaining enzymes, preparing them for use in papermaking (Azeez, 2018). Biopulping can be more time‐consuming and expensive compared to traditional pulping methods. Therefore, it is crucial to strike the right balance between sustainability and cost‐effectiveness (Kumar et al., 2020). Cold biopulping employs enzymes like pectinases, hemicellulases, and cellulases to break down pectin, hemicellulose, and cellulose, respectively. The utilization of cold enzymes in biopulping has demonstrated the potential to reduce pulping time, increase pulp yield, and potentially enhance the brightness and strength of the final product (Hamid et al., 2022). One of the primary advantages of using cold‐active enzymes in biopulping is their capacity to reduce the energy requirements of the pulping process, making it a more sustainable and eco‐friendly choice. Additionally, these cold‐active enzymes including cellulases and hemicellulases can enhance the yield and quality of the final pulp product. For example, cellulases and hemicellulases break down cellulose and hemicellulose into smaller sugars, which can be used as an energy source or as raw materials for other processes, leading to improved paper strength and brightness (Wei et al., 2021).

While biopulping holds immense potential, there are several challenges that must be addressed before it can be widely adopted in the industry. Key challenges include scaling up the process for commercial production, improving the efficiency and speed of the pulping process, and reducing associated costs (Kumar et al., 2020). Despite these challenges, some companies have already begun to integrate biopulping into their operations. Startups and research institutions, for instance, are exploring the use of fungi and enzymes to break down lignin in wood chips (Goodell et al., 2020). Established companies in the paper and pulp industry are also investing in biopulping research and development with the aim of bringing this technology to the market. Additionally, the utilization of cold enzymes in biopulping remains a dynamic area of research, with ongoing efforts to optimize the enzymes and their application conditions to maximize effectiveness and efficiency (Wei et al., 2021). Microbial cold‐active enzymes are often used in biopulping because they are effective at breaking down lignin at low temperatures, which results in less energy consumption and a lower carbon footprint compared to traditional pulping methods (Joseph et al., 2008). In addition, microbial enzymes are biodegradable and non‐toxic, making them a more sustainable alternative to chemical pulping methods (Kumar & Rani, 2019).

Enzymatic biobleaching is a highly effective technique that supports the breakdown of hemicellulose and aids in the removal of lignin. Microbial xylanases are utilized in biobleaching to enhance the extractability of lignin in subsequent phases without directly damaging it. These xylanases target the lignin‐carbohydrate complex, leading to the removal and modification of xylan and glucomannan structures, resulting in improved delignification efficiency (Çiçekler & Tutuş, 2019; Colonia et al., 2019; Zakaria et al., 2015). Xylanases, among various enzymes, exhibit great potential in the pulp and paper industries. They interact with xylan in wood, breaking down lignin‐carbohydrate linkages, thereby enhancing paper quality when applied in biopulping and biobleaching (Gupta et al., 2013; Pandey et al., 2022). Cold‐active xylanases play a crucial role in various low‐temperature processes, including biopulping, biobleaching, and applications in clarifying fruit juices and textiles. Their high catalytic activity at lower temperatures makes them excellent biocatalysts (Collins et al., 2005; Suhaib et al., 2018). These enzymes find efficient use in diverse industrial and biotechnological processes, spanning molecular biology, food and beverages, detergent production, medical applications, paper manufacturing, and textiles (Hamid, 2021; Wackett, 2019).

Xylanases are particularly valuable in reducing the need for oxidizing chemicals by approximately 20%, resulting in improved brightness during biobleaching processes. This is attributed to their bleach‐boosting properties (Bajaj & Mahajan, 2019; Garg et al., 2011; Shrinivas et al., 2010). One limitation of enzyme utilization in biopulping is their limited penetration into wood chips, making their effectiveness somewhat challenging. To address this, enzymes have historically been applied to partially defibrated raw wood material or during the bleaching of chemical pulp (Burton, 2001; Eugenio et al., 2010; Lehr et al., 2021). Alternatively, the use of vacuum or pressure can expedite the migration of enzymes into wood chips (Maijala et al., 2008). Another essential enzyme in the paper industry is endo‐β‐mannanase. This enzyme hydrolyses linear glucomannan, mannan, and 1,4‐glycosidic linkages within the mannan chain in a random manner. The resulting hydrolysate, composed of mannan‐oligosaccharides, must undergo conversion to monosaccharides through the action of the enzyme mannosidase (Hlalukana et al., 2021). Enzyme‐treated fibres may exhibit a slight reduction in inter‐fibre bonding strength when cellulose is present, but this does not compromise the mechanical strength of the fibres (Valls et al., 2010). In the absence of cellulose, xylanase application leads to increased viscosity, while hemicellulose hydrolysis eases lignin removal (Li et al., 2010; Walia et al., 2017).

The application of psychrozymes in biopulping represents a promising advancement within the paper and pulp industry. These microbial cold enzymes have the potential to play a significant role in the industry's future by reducing the energy requirements for pulping and providing a more environmentally friendly alternative to traditional pulping methods (Wei et al., 2021). Cold enzymes play a vital role in the biopulping process by breaking down lignocellulosic materials in wood and agricultural waste into smaller components. Cold enzymes enable the breakdown of lignin, cellulose, and hemicellulose at lower temperatures compared to conventional pulping processes, which typically involve high temperatures and chemical treatments (Kumar & Rani, 2019). However, the utilization of cold enzymes in biopulping may pose some challenges, such as the requirement for high enzyme concentrations and extended reaction times. Additionally, the cost associated with producing and purifying cold enzymes can limit their widespread adoption in industry. It is crucial to note that while biopulping aims to reduce the use of chemicals in the pulping process, there may still be situations where chemicals are used, but their application should be minimal and rigorously controlled to ensure that the environmental benefits of biopulping are not compromised. The use of chemicals should be transparent, and their environmental impact should be thoroughly assessed (Kumar et al., 2020). Biopulping has the potential to play a pivotal role in the future of the paper and pulp industry. Despite the challenges that need to be addressed, the potential advantages offered by cold‐active enzymes in biopulping make it a promising avenue for ongoing research and development. The mesophilic enzymes used in biopulping such as xylanase for xylan degradation (Walia et al., 2017), laccase for lignin degradation (Zerva et al., 2019), cellulase for cellulose degradation (Li et al., 2019), and pectinase for pectin degradation (Haile & Ayele, 2022) may be replaced by cold‐active xylanase, laccase, cellulase, and pectinase, respectively; that improve pulp and paper quality and also decreases energy demands. Research efforts are continuing to optimize cold enzymes and their application in biopulping, making them an attractive option for the future of the pulp and paper industry (Mesbah, 2022).

## COLD‐ACTIVE ENZYMES FOR ENVIRONMENTAL BIOREMEDIATION IN COLD CLIMATES

Environment is contaminated by various activities including industrial, tourist activities, human migration, transportation etc. that become a serious threat to human populations and create serious environmental hazards. Effective solution for this problem is bioremediation due to less destruction and economic benefits. Enzymes are highly efficient green biocatalysts with huge energy savings, reducing chemical reactions, non‐toxic, biodegradable, safe, cost‐efficient, and ecofriendly (Bilal et al., 2019). Psychrophilic microorganisms and its cold‐active enzymes are gaining attention due to its activity under harsh conditions and suitability in industrial and biotechnological application with low activation energy. Majority of the Earth's portion experiences cold climates ranging from Antarctica to Arctic, high mountains to deep sea, lakes etc., that are subjected to contamination (Rota et al., 2022). Microorganisms in these regions play an important role in nutrient recycling, mineralization and biodegradation with the help of enzymes adapted to such extreme conditions. Pollution in cold regions is increasing tremendously with petroleum hydrocarbons, chlorinated chemicals, solvents, pesticides, etc. These pollutants remain much longer in cold regions because of low bioavailability, lack of microbial populations adapted to low temperature and harsh climatic conditions. Several factors also affect the process such as properties of the pollutant, metabolic limitations, mass transfer through cell membrane, change in temperature, bioavailability, oxygen, electron acceptors, toxicity, and freeze‐thaw process (Yang et al., 2009). In cold regions, the biodegradation is affected by low temperature, affecting the physical nature that reduces the bioavailability of the microbial community. Further, the increased viscosity and decreased volatility affect the biodegradation (Bajaj & Singh, 2015). The higher specificity and catalytic activity at low temperature makes cold‐active enzymes appropriate for bioremediation (Kumar et al., 2021). Bioremediation in cold regions, contaminated with certain pollutants, are carried out by bioaugmentation methods. There are certain limitations for these strategies including low nutrient availability, poor availability of pollutants to the microbial populations and efficiency of the strain to degrade particular chemicals. To overcome these limitations a thorough understanding of bioremediation of pollutants in cold regions is needed. Several studies were conducted to implement bioremediation strategies in the cold regions by using psychrophilic microbial consortia, cold‐active enzymes and recombinant bioaugmentation (Chaudhary & Kim, 2019; Davoodi et al., 2020).

Many of the cold‐active enzymes producing bacteria and fungi were reported for the bioremediation process. Due to the increasing pollution and climatic variations, the number of microbes is decreasing in temperate regions. Inoculations of consortia‐producing cold‐active enzymes helped in boosting the degradation of hydrocarbons (Gerday et al., 2000). However, for minimizing the environmental contamination and for bioremediation in cold regions a huge economical input is required to achieve rapid and successful results. The degree of success in bioremediation in cold regions is affected during seasonal transition. Cold‐adapted microorganisms in appropriate populations that are tolerant to harsh environmental conditions perform biodegradation at in‐situ conditions for the removal of pollutants (Miri et al., 2022). The efficient biodegradation with cold‐active enzymes acquired by structural adaptation of proteins through genetic modifications and long‐term selection process (Struvay & Feller, 2012). The variation in amino acid compared to mesophilic enzymes makes weaker interaction in protein making it flexible at low temperature (Feller, 2010). Lowering the activation energy because of weak substrate binding subsequently increases the rate of the reaction. Thus psychrophilic microorganisms and cold‐active enzymes play a major role in bioremediation of pollutants in cold regions compared to mesophilic counterparts.

Bioremediation in Arctic and sub‐Arctic regions has been achieved successfully, but removal of pollutants from the cold regions faces many challenges compared to other regions. To preserve the environment there is a need to reconsider, adapt and improve the current strategies in cold environments. Therefore, In Situ methods such as bioaugmentation and biostimulation are adopted. Bioaugmentation in cold regions is the addition of cold‐adapted microorganisms capable of degrading a particular pollutant to speed up the rate of biodegradation (Yuan et al., 2018). Studies were conducted to determine the effect of bioaugmentation in polluted areas of cold regions (Filler et al., 2008; Hosokawa et al., 2011). In cold regions, bioaugmentation becomes successful when the native population capable of degrading pollutants is low (Camenzuli & Freidman, 2015; Patel et al., 2018). Biostimulation is another process by which nutrients are supplied to soil to stimulate the native microbial population. The presence of native degrading microorganisms enriched bioremediation in cold regions contaminated with hydrocarbons (Simpanen et al., 2016). A pre‐optimized biostimulation process removed 75% of the pollutants in 40 days (Martínez Álvarez et al., 2017). Another method of bioremediation is addition of free water that enhances the microbial load in soil. Only a few reports are available on this method due to the lesser efficiency compared to the above‐mentioned two methods (Stallwood et al., 2005). The increased proportion of microbial load in bioaugmentation and bio‐stimulation degrades the pollutant effectively (Kuhn et al., 2009).

## COLD‐ACTIVE ENZYMES IN BIOTRANSFORMATION AND MOLECULAR BIOLOGY APPLICATIONS

Cold‐active enzymes play a pivotal role in biotransformation, particularly in frigid environments such as the Arctic and Antarctic regions (Peng et al., 2022). These enzymes exhibit functionality at low temperatures, where conventional enzymes would remain inactive, rendering them valuable tools in biocatalysis and bioprocessing at low temperatures. Moreover, the utilization of cold‐active enzymes in biotransformation offers several advantages, including decreased energy consumption, reduced production costs, and enhanced reaction efficiency (Jin et al., 2019). Furthermore, the integration of cold‐active enzymes into biotransformation processes contributes to a reduction in environmental impacts by curbing waste generation and diminishing the overall carbon footprint of the operation. Consequently, the study and application of cold‐active enzymes in biotransformation have emerged as a critical area of research with the potential to revolutionize diverse industries, such as food processing, pharmaceuticals, and biofuels (Carrasco et al., 2012; Gupta et al., 2020). Cold‐active lipases find applications in bioremediation, biotransformation, baking, food processing, as well as in molecular biology and the detergent sector (Joseph et al., 2008). In summary, the role of cold‐active enzymes in biotransformation holds promise for sustainable and efficient bioprocessing and biocatalysis, particularly in frigid environments. For instance, cold‐active chitinase enzymes and certain yeast psychrozymes are employed in the conversion of chitin into bioethanol (Dahiya et al., 2006). Future research endeavours in this domain may encompass the exploration and optimization of novel cold‐active enzymes, alongside the development of innovative biotransformation processes that fully harness the unique attributes of these enzymes.

Cold‐active enzymes are characterized by their ability to remain active at low temperatures while being vulnerable to heat, making them valuable assets in various fields including molecular biology (Cavicchioli et al., 2011; Kuddus, 2018; Marx et al., 2007; Sahay et al., 2013). Microbial enzymes possess a range of distinctive properties, such as stability, eco‐friendliness, high productivity, flexibility, and economic viability, among others. These attributes have amplified the importance of microbial enzymes across multiple industries (Gurung et al., 2013; Mangiagalli & Lotti, 2021). Various researchers have reported on the application and potential of cold‐active enzymes in molecular biology and food biotechnology (Hamid, 2021; Hamid et al., 2022; Kuddus, 2018). The heat sensitivity associated with cold‐adapted enzymes is a critical feature for sequential enzymatic processes used in various molecular biology techniques (Kumar et al., 2021). In molecular biology, alkaline phosphatases (AP) are widely used, including in cloning, where they are employed to dephosphorylate the 5′ end of a linearized DNA fragment, preventing its recircularization (Sharma et al., 2014). Traditionally, the only available APs were derived from *E*. *coli* or calf intestinal tissue and required heat‐resistant deactivation with detergents, which could potentially interfere with subsequent processes. In contrast, psychrophilic heat‐labile APs can be quickly inactivated in the same tube by subjecting them to a mild heat treatment at 65°C (Hamdan, 2018). The first heat‐labile AP was discovered in a bacterium from Antarctica and was purified in 1984. Commercially produced as Antarctic phosphatase by New England Biolabs, it was initially isolated from another Antarctic bacterium and further improved through directed evolution (Koutsioulis et al., 2008). In 1993, ArcticZymes in Tromsø, Norway, introduced the first commercially available cold‐adapted AP, using the Arctic shrimp Pandalus borealis. In 2010, ArcticZymes unveiled a genetically engineered version of this AP. Recent metagenomics library creation from ocean‐tidal flat sediments on the west coast of Korea led to the discovery of a novel psychrophilic AP (Lee et al., 2015) with properties and effectiveness similar to other commercially available APs. Numerous approaches and techniques have been developed for in‐depth studies of the structural and molecular aspects of cold‐active lipases (Do et al., 2013; Jeon et al., 2009; Parra et al., 2008).

In molecular biology, cold‐adapted enzymes find further applications: (a) Uracil‐DNA N‐glycosylase (UNG) catalyses the release of free uracil from uracil‐con (Schormann et al., 2014). UNG is employed in various applications, including preventing carry‐over contamination in PCR and RT PCR, site‐directed mutagenesis, and SNP genotyping (Tetzner, 2009). ArcticZymes offers a commercially available cold‐adapted UNG enzyme derived from Atlantic cod (*Gadus morhua*) through recombinant DNA technology. This enzyme is entirely and irreversibly inactivated by heat treatment at 55°C (Lanes et al., 2002). Another heat‐labile UNG was discovered in the genome of the bacterium *Psychrobacter* sp. HJ147, and it was cloned and expressed using *E*. *coli*. It functions optimally between 20 and 25°C and has a half‐life of two minutes at 40°C (Lee et al., 2009). New England Biolabs provides an Antarctic Thermolabile UDG, a recombinant UNG enzyme created in *E*. *coli* from a psychrophilic marine bacterium, which remains active at temperatures above 50°C. (b) Double‐strand‐specific DNase can digest double‐stranded DNA without affecting single‐stranded DNA molecules like primers or probes. This enzyme is used for genomic DNA extraction from RNA preparations or PCR cleanup (Rittié & Perbal, 2008). ArcticZymes offers a heat‐labile version of this enzyme, originally isolated from shrimp (Pandalus borealis) and subsequently genetically modified to become inactive at 55°C. Affymetrix USB (Santa Clara, CA, USA) provides a recombinant version produced in Pichia pastoris, which is inactivated after exposure to 70°C for 25–30 min. (c) Cryonase, a recombinant cold‐active nuclease from a psychrophilic strain of *Shewanella* sp, is available from Takara‐Clontech (Mountain View, CA, USA). This enzyme, created in E. coli, can digest any form of DNA or RNA, including single‐stranded, double‐stranded, linear, or circularized molecules (Bruno et al., 2019). Cryonase remains active even when samples are kept on ice and can be completely inactivated with a 30‐min incubation at 70°C.

Nucleases play a crucial role in breaking down DNA or RNA and are essential for removing contaminating nucleic acids from the reaction mixture (Yang, 2011). However, after their activity, it is necessary to eliminate the nucleases from the reaction mixture. Typically, this is achieved by heating the enzyme mixture to deactivate it. In such cases, the use of cold‐adapted nucleases offers advantages as they can be rapidly and effectively inactivated by mild heat treatments. Cold‐adapted nucleases sourced from psychrophilic microorganisms, including *Pandalus borealis* and *Shewanella* sp., are commercially available (Nandanwar et al., 2020). Furthermore, the exploration of a cold‐adapted nuclease derived from a psychrophilic strain of *Psychromonas ingrahamii* for potential applications in molecular biology is promising (Maciejewska et al., 2019). Another study identified a cold‐adapted RNase enzyme from *Psychrobacter*, a bacterium found in Antarctic Sea ice, opening up new avenues for research and study in molecular biology (Wang et al., 2019). Ligases, on the other hand, facilitate the formation of phosphodiester links between two DNA fragments, allowing the joining of these DNA fragments. Bacteriophages are commonly used as a source of ligases for this purpose. In molecular biology, the optimal temperature for the activity of DNA ligases is often low, making cold‐adapted ligases advantageous (Nandanwar et al., 2020). Recent research has described three cold‐adapted DNA ligases from psychrophilic species, highlighting their optimal temperatures and thermal stabilities. Further studies may explore their applications in molecular biology (Berg et al., 2019).

## COLD‐ACTIVE ENZYMES: SUSTAINABLE SOURCE FOR BIO‐BASED ECONOMY

Cold‐active enzymes play a crucial role in the bio‐based economy by enabling biotechnological processes to occur at low temperatures. This is significant because many industrial processes traditionally require high temperatures, which consume more energy and may not be suitable for temperature‐sensitive materials or organisms. Cold‐active enzymes offer several advantages in various sectors of the bio‐based economy.

Cold‐active enzymes function optimally at low to moderate temperatures (often below 50°C), reducing the energy required for heating in industrial processes. This contributes to the overall sustainability of bio‐based production systems with energy efficiency. It involves the preservation of heat‐sensitive compounds. In industries like food, pharmaceuticals, and cosmetics, cold‐active enzymes can be used to process and manufacture products without compromising the integrity of heat‐sensitive compounds (Kuddus et al., 2011). This is crucial for preserving the quality and functionality of these products. As for environmental impact, lowering the temperature requirements for industrial processes reduces greenhouse gas emissions, as less energy is needed for heating. This aligns with the goals of a more sustainable and environmentally friendly bio‐based economy. Cold‐active enzymes are employed in environmental bioremediation processes also. They can help clean up cold environments, such as polar regions, where traditional enzymes might be less effective due to low temperatures. Cold‐active enzymes are also used in cold food processing where it can be used to improve processes like cheese production, beer brewing, and yogurt making, all of which require low‐temperature fermentation or maturation. In biofuel production, enzymes used in the production of biofuels from lignocellulosic biomass often work more efficiently at lower temperatures. Cold‐active enzymes can play a role in breaking down lignocellulose into fermentable sugars for bioethanol or biogas production. It can play a significant role in biorefineries for the efficient conversion of biomass into biofuels and other valuable chemicals.

Cold‐active enzymes also have significant impact in medical and pharmaceutical applications. These enzymes are valuable in biopharmaceutical production processes, including the expression and purification of temperature‐sensitive proteins and the production of vaccines and biologics. Cold‐active enzymes along with its producing organisms can be applied in wastewater treatment systems in regions with colder climates, helping to degrade pollutants and organic matter more efficiently. In addition, it used in aquaculture, such as those in fish feed, may benefit from cold‐active enzymes as they allow for optimal digestion in the lower‐temperature environments of fish tanks or open water systems. Cold‐active enzymes are of interest to researchers and innovators in biotechnology and synthetic biology for their unique properties. Scientists can explore their potential in novel applications and develop new biotechnological processes. In summary, cold‐active enzymes are essential components of the bio‐based economy, contributing to increased sustainability, reduced energy consumption, and the expansion of biotechnological applications in various sectors. Their unique ability to function at low temperatures opens up new possibilities for efficient, environmentally friendly processes and products.

## CONCLUSION AND FUTURE PROSPECTS OF COLD‐ACTIVE ENZYMES

The scope of cold‐active enzymes is promising, as they continue to gain importance in various industries and applications. Their ability to function at lower temperatures can enhance the efficiency of enzymatic hydrolysis and fermentation processes. In pharmaceutical and biopharmaceutical production processes, they can aid in the expression, purification, and formulation of temperature‐sensitive proteins and biologics, improving yields and product quality. Cold‐active enzymes will continue to be utilized in the food and beverage industry to improve the quality and processing of products like dairy and beverages. In regions with cold climates, cold‐active enzymes can be employed in environmental cleanup efforts, such as the remediation of oil spills and other pollutants in cold‐water ecosystems. It can contribute to more efficient feed conversion in aquaculture, leading to improved sustainability and reduced environmental impact. In agriculture, these enzymes may be applied to enhance nutrient availability and soil health in colder climates. As research into extremophiles advances, cold‐active enzymes from these organisms could be used in various industrial applications, including those requiring low temperatures. Advances in enzyme engineering techniques could lead to the creation of tailor‐made cold‐active enzymes with enhanced properties, stability, and specificity for specific applications. Cold‐active enzymes might find applications in medical research and diagnostics, potentially assisting in the development of novel diagnostic assays and therapies that require low‐temperature conditions. It could play a role in enhancing the efficiency of cold‐chain logistics, ensuring the preservation and quality of temperature‐sensitive products during storage and transportation. Ongoing research into the molecular mechanisms and properties of cold‐active enzymes could uncover new insights that drive innovation in various fields, potentially leading to breakthroughs in areas like enzyme stability, activity, and substrate specificity. Advances in bioinformatics and metagenomics could lead to the discovery of novel cold‐active enzymes from unexplored environments, further expanding the enzyme toolbox for various applications. Overall, the future prospects of cold‐active enzymes are closely tied to the growing demand for sustainable, energy‐efficient, and environmentally friendly solutions across industries.

## AUTHOR CONTRIBUTIONS

**Mohammed Kuddus:** Conceptualization; resources; supervision; writing – original draft; writing – review and editing. **Roohi:** Resources; writing – original draft; writing – review and editing. **Naushin Bano:** Data curation; resources; writing – original draft. **Gouse Basha Sheik:** Data curation; resources; writing – original draft. **Babu Joseph:** Conceptualization; resources; writing – original draft; writing – review and editing. **Burhan Hamid:** Data curation; resources; writing – original draft. **Raveendran Sindhu:** Resources; writing – review and editing. **Aravind Madhavan:** Supervision; writing – review and editing.

## FUNDING INFORMATION

No funding information provided.

## CONFLICT OF INTEREST STATEMENT

There is no conflict of interest.

## References

[mbt214467-bib-0001] Abdella, B. , Youssif, A.M. , Sabry, S.A. & Ghozlan, H.A. (2023) Production, purification, and characterization of cold‐active lipase from the psychrotroph *pseudomonas* sp. A6. Brazilian Journal of Microbiology, 54, 1623–1633. 37531003 10.1007/s42770-023-01079-y PMC10484855

[mbt214467-bib-0002] Adapa, V. , Pulicherla, K. & Rao, K.R.S.S. (2019) Marine psychrophile‐derived cold‐active polygalacturonase: enhancement of productivity in *Thalassospira frigidphilosprofundus* S3BA12 by whole cell immobilization. Biochemical Engineering Journal, 144, 135–147.

[mbt214467-bib-0003] Adapa, V. , Ramya, L. , Pulicherla, K. & Rao, K.R.S.S. (2014) Cold active pectinases: advancing the food industry to the next generation. Applied Biochemistry and Biotechnology, 172, 2324–2337. 24390855 10.1007/s12010-013-0685-1

[mbt214467-bib-0004] Alam, S.I. , Dixit, A. , Reddy, G.S.N. , Dube, S. , Palit, M. , Shivaji, S. et al. (2006) *Clostridium schirmacherense* sp. nov., an obligatory anaerobic, proteolytic, psychrophilic bacterium isolated from lake sediment of Schirmacher oasis, Antarctica. International Journal of Systematic and Evolutionary Microbiology, 56(4), 715–720. 16585682 10.1099/ijs.0.63808-0

[mbt214467-bib-0006] Al‐Ghanayem, A.A. & Joseph, B. (2020) Current prospective in using cold‐active enzymes as eco‐friendly detergent additive. Applied Microbiology and Biotechnology, 104, 2871–2882. 32037467 10.1007/s00253-020-10429-x

[mbt214467-bib-0009] Azeez, M.A. (2018) Pulping of non‐woody biomass. In: Pulp and paper processing. London, United Kingdom: InTech.

[mbt214467-bib-0011] Bajaj, P. & Mahajan, R. (2019) Cellulase and xylanase synergism in industrial biotechnology. Applied Microbiology and Biotechnology, 103(21), 8711–8724. 31628521 10.1007/s00253-019-10146-0

[mbt214467-bib-0012] Bajaj, S. & Singh, D.K. (2015) Biodegradation of persistent organic pollutants in soil, water and pristine sites by cold‐adapted microorganisms: mini review. International Biodeterioration and Biodegradation, 100, 98–105.

[mbt214467-bib-0013] Bajpai, P. (2018) Biopulping. In: Biotechnology for pulp and paper processing, pp. 113–147. New York: Springer.

[mbt214467-bib-0014] Bakermans, C. , Ayala‐Del‐Río, H.L. , Ponder, M.A. , Vishnivetskaya, T. , Gilichinsky, D. , Thomashow, M.F. et al. (2006) *Psychrobacter cryohalolentis* sp. nov. and *Psychrobacter arcticus* sp. nov., isolated from Siberian permafrost. International Journal of Systematic and Evolutionary Microbiology, 56(6), 1285–1291. 16738105 10.1099/ijs.0.64043-0

[mbt214467-bib-0016] Barghini, P. , Moscatelli, D. , Garzillo, A.M.V. , Crognale, S. & Fenice, M. (2013) High production of cold‐tolerant chitinases on shrimp wastes in bench top bioreactor by the Antarctic fungus *Lecanicillium muscarium* CCFEE 5003: bioprocess optimization and characterization of two main enzymes. Enzyme and Microbial Technology, 53, 331–338. 24034432 10.1016/j.enzmictec.2013.07.005

[mbt214467-bib-0017] Barroca, M. , Santos, G. , Gerday, C. & Collins, T. (2017) Biotechnological aspects of cold‐active enzymes. In: Psychrophiles: from biodiversity to biotechnology, pp. 461–475. Berlin, Heidelberg: Springer.

[mbt214467-bib-0018] Bashir, I. , Lone, F.A. , Bhat, R.A. , Mir, S.A. , Dar, Z.A. & Dar, S.A. (2020) Concerns and threats of contamination on aquatic ecosystems. In: Bioremediation and biotechnology: sustainable approaches to pollution degradation, pp. 1–26. Cham: Springer.

[mbt214467-bib-0019] Benforte, F.C. , Colonnella, M.A. , Ricardi, M.M.S. , Venero, E.C. , Lizarraga, L. , Lopez, N.I. et al. (2018) Novel role of the LPS core glycosyltransferase WapH for cold adaptation in the Antarctic bacterium *Pseudomonas extremaustralis* . PLoS One, 13, e0192559. 29415056 10.1371/journal.pone.0192559 PMC5802925

[mbt214467-bib-0020] Benmrad, M.O. , Moujehed, E. , Elhoul, M.B. , Mechri, S. , Bejar, S. et al. (2018) Production, purification, and biochemical characterization of serine alkaline protease from *Penicillium chrysogenium* strain X5 used as excellent bio‐additive for textile processing. International Journal of Biological Macromolecules, 119, 1002–1016. 30081129 10.1016/j.ijbiomac.2018.07.194

[mbt214467-bib-0021] Berg, K. , Leiros, I. & Williamson, A. (2019) Temperature adaptation of DNA ligases from psychrophilic organisms. Extremophiles, 2019(23), 305–317. 10.1007/s00792-019-01082-y 30826937

[mbt214467-bib-1000] Bhardwaj, S. , Bhardwaj, N.K. & Negi, Y.S. (2019) Cleaner approach for improving the papermaking from agro and hardwoodblended pulps using biopolymers. Journal of Cleaner Production, 213, 134–142.

[mbt214467-bib-0023] Bhat, A. , Riyaz‐Ul‐Hassan, S. , Ahmad, N. , Srivastava, N. & Johri, S. (2013) Isolation of cold‐active, acidic endocellulase from Ladakh soil by functional metagenomics. Extremophiles, 17, 229–239. 23354361 10.1007/s00792-012-0510-8

[mbt214467-bib-0024] Bhatia, R.K. , Ullah, S. , Hoque, M.Z. , Ahmad, I. , Yang, Y.H. , Bhatt, A.K. et al. (2021) Psychrophiles: a source of cold‐adapted enzymes for energy efficient biotechnological industrial processes. Journal of Environmental Chemical Engineering, 9(1), 104607.

[mbt214467-bib-0025] Białkowska, A. & Turkiewicz, M. (2014) Cold‐adapted yeasts. Springer, pp. 377–395. Berlin, Heidelberg.

[mbt214467-bib-0026] Bilal, M. , Adeel, M. , Rasheed, T. , Zhao, Y. & Iqbal, H.M.N. (2019) Emerging contaminants of high concern and their enzyme‐assisted biodegradation – a review. Environment International, 124, 336–353. 30660847 10.1016/j.envint.2019.01.011

[mbt214467-bib-0027] Bruno, S. , Coppola, D. , Di Prisco, G. , Giordano, D. & Verde, C. (2019) Enzymes from marine polar regions and their biotechnological applications. Marine Drugs, 17, 1–36. 10.3390/md17100544 PMC6835263 31547548

[mbt214467-bib-2000] Buisman, G.J. , Van Helteren, C.T. , Kramer, G.F. , Veldsink, J.W. , Derksen, J.T. & Cuperus, F.P. (1998) Enzymatic esterifications of functionalized phenols for the synthesis of lipophilic antioxidants. Biotechnology Letters, 20, 131–136.

[mbt214467-bib-0028] Burton, S.W. (2001) Low energy thermomechanical pulping process using an enzyme treatment between refining zones. Patent US 6267841.

[mbt214467-bib-0029] Camenzuli, D. & Freidman, B.L. (2015) On‐site and in situ remediation technologies applicable to petroleum hydrocarbon contaminated sites in the Antarctic and Arctic. Polar Research, 34(1), 24492.

[mbt214467-bib-0030] Canion, A. , Prakash, O. , Green, S.J. , Jahnke, L. , Kuypers, M.M. & Kostka, J.E. (2013) Isolation and physiological characterization of psychrophilic denitrifying bacteria from permanently cold Arctic fjord sediments (Svalbard, Norway). Environmental Microbiology, 15(5), 1606–1618. 23530773 10.1111/1462-2920.12110

[mbt214467-bib-0031] Carrasco, M. , Rozas, J.M. , Barahona, S. , Alcaíno, J. , Cifuentes, V. & Baeza, M. (2012) Diversity and extracellular enzymatic activities of yeasts isolated from King George Island, the sub‐Antarctic region. BMC Microbiology, 12, 251. 23131126 10.1186/1471-2180-12-251 PMC3499239

[mbt214467-bib-0032] Cavicchioli, R. , Charlton, T. , Ertan, H. , Mohd Omar, S. , Siddiqui, K.S. & Williams, T.J. (2011) Biotechnological uses of enzymes from psychrophiles. Microbial Biotechnology, 4, 449–460. 21733127 10.1111/j.1751-7915.2011.00258.x PMC3815257

[mbt214467-bib-0033] Chaudhary, D.K. & Kim, J. (2019) New insights into bioremediation strategies for oil‐contaminated soil in cold environments. International Biodeterioration and Biodegradation, 142, 58–72.

[mbt214467-bib-0035] Çiçekler, M. & Tutuş, A. (2019) Effects of xylanase enzyme on oxygen bleaching of wheat straw pulp. 2. International Mediterranean Symposium, 23–25 May, Mersin, Turkey, pp. 359–368.

[mbt214467-bib-0036] Collins, T. , Gerday, C. & Feller, G. (2005) Xylanases, xylanase families and extremophilic xylanases. FEMS Microbiology Reviews, 29, 3–23. 15652973 10.1016/j.femsre.2004.06.005

[mbt214467-bib-0038] Colonia, B.S.O. , Woiciechowski, A.L. , Malanski, R. , Letti, L.A.J. & Soccol, C.R. (2019) Pulp improvement of oil palm empty fruit bunches associated to solid‐state biopulping and biobleaching with xylanase and lignin peroxidase cocktail produced by *Aspergillus* sp. LPB‐5. Bioresource Technology, 285, 121361. 31018172 10.1016/j.biortech.2019.121361

[mbt214467-bib-0039] Dahiya, N. , Tewari, R. & Hoondal, G.S. (2006) Biotechnological aspects of chitinolytic enzymes: a review. Applied Microbiology and Biotechnology, 71, 773–782. 16249876 10.1007/s00253-005-0183-7

[mbt214467-bib-0040] Davoodi, S.M. , Miri, S. , Taheran, M. , Brar, S.K. , Galvez‐Cloutier, R. & Martel, R. (2020) Bioremediation of unconventional oil contaminated ecosystems under natural and assisted conditions: a review. Environmental Science & Technology, 54(4), 2054–2067. 31904944 10.1021/acs.est.9b00906

[mbt214467-bib-0041] Dewan, S. (2017) Global markets for enzymes in industrial applications. Massachusetts, USA: BCC Research LLC.

[mbt214467-bib-0042] Dhanjal, D.S. , Singh, S. , Kumar, V. , Ramamurthy, P.C. , Chopra, C. , Wani, A.K. et al. (2023) Isolation and characterization of cellulase‐producing Myxobacterial strain from the unique niche of Mirgund wetland from the North‐Western Himalayas. Journal of Applied Biology and Biotechnology, 11(5), 119–125.

[mbt214467-bib-0043] Divya, K. & Padma, P.N. (2015) Use of Plackett‐Burman design for rapid screening of diverse raw pectin sources for cold‐active polygalacturonase and amylase production by *Geotrichum* sp. International Journal of Current Microbiology and Applied Sciences, 4(6), 821–827.

[mbt214467-bib-0044] Do, H. , Lee, J.H. , Kwon, M.H. , Song, H.E. , An, J.Y. , Eom, S.H. et al. (2013) Purification, characterization and preliminary X‐ray diffraction analysis of a cold‐active lipase (CpsLip) from the psychrophilic bacterium *Colwellia psychrerythraea* 34H. Acta Crystallographica Section F: Structural Biology Communications, 69, 920–924. 10.1107/S1744309113019428 PMC3729175 23908044

[mbt214467-bib-0047] Duarte, A.W.F. , Dos Santos, J.A. , Vianna, M.V. , Vieira, J.M.F. , Mallagutti, V.H. , Inforsato, F.J. et al. (2018) Cold‐adapted enzymes produced by fungi from terrestrial and marine Antarctic environments. Critical Reviews in Biotechnology, 38(4), 600–619. 29228814 10.1080/07388551.2017.1379468

[mbt214467-bib-0048] Eugenio, M.E. , Santos, S.M. , Carbajo, J.M. , Martín, J.A. , Martín‐Sampedro, R. , González, A.E. et al. (2010) Kraft pulp biobleaching using an extracellular enzymatic fluid produced by *Pycnoporus sanguineus* . Bioresource Technology, 101, 1866–1870. 19857961 10.1016/j.biortech.2009.09.084

[mbt214467-bib-0049] Farooq, S. , Nazir, R. , Ganai, S.A. & Ganai, B.A. (2021) Isolation and characterization of a new cold‐active protease from psychrotrophic bacteria of Western Himalayan glacial soil. Scientific Reports, 11, 12768. 34140593 10.1038/s41598-021-92197-w PMC8211794

[mbt214467-bib-0050] Farrell, J. & Rose, A.H. (1967) Temperature effects on microorganisms. In: Rose, A.H. (Ed.) Thermobiology, Vol. 21. London: Academic, pp. 147–218.

[mbt214467-bib-0051] Feller, G. (2010) Protein stability and enzyme activity at extreme biological temperatures. Journal of Physics. Condensed Matter: An Institute of Physics Journal, 22(32), 323101. 21386475 10.1088/0953-8984/22/32/323101

[mbt214467-bib-0052] Feller, G. & Gerday, C. (2003) Psychrophilic enzymes: hot topics in cold adaptation. Nature Reviews Microbiology, 1(3), 200–208. 15035024 10.1038/nrmicro773

[mbt214467-bib-0053] Feller, G. , Le Bussy, O. & Gerday, C. (1998) Expression of psychrophilic genes in mesophilic hosts: assessment of the folding state of a recombinant α‐amylase. Applied and Environmental Microbiology, 64, 1163–1165. 9501457 10.1128/aem.64.3.1163-1165.1998 PMC106386

[mbt214467-bib-0054] Ferraz, A. , Guerra, A. , Mendonça, R. , Masarin, F. , Vicentim, M.P. , Aguir, A. et al. (2008) Technological advances and mechanistic basis for fungal biopulping. Enzyme and Microbial Technology, 43, 178–185.

[mbt214467-bib-0055] Filler, D.M. , Snape, I. & Barnes, D.L. (Eds.). (2008) Bioremediation of petroleum hydrocarbons in cold regions. Cambridge: Cambridge University Press.

[mbt214467-bib-0056] Furhan, J. (2020) Adaptation, production, and biotechnological potential of cold‐adapted proteases from psychrophiles and psychrotrophs: recent overview. Journal, Genetic Engineering & Biotechnology, 18(1), 36. 10.1186/s43141-020-00053-7 PMC7387391 32725297

[mbt214467-bib-0057] Garg, G. , Dhiman, S.S. , Mahajan, R. , Kaur, A. & Sharma, J. (2011) Bleach boosting effect of crude xylanase from *Bacillus stearothermophilus* SDX on wheat straw pulp. New Biotechnology, 28(1), 58–64. 20709630 10.1016/j.nbt.2010.07.020

[mbt214467-bib-0058] Garg, G. , Singh, A. , Kaur, A. , Singh, R. , Kaur, J. & Mahajan, R. (2016) Microbial pectinases: an ecofriendly tool of nature for industries. 3 Biotech, 6, 1–13. 10.1007/s13205-016-0371-4 PMC4746199 28330117

[mbt214467-bib-0059] Gerday, C. (2013) Psychrophily and catalysis. Biology, 2, 719–741. 24832805 10.3390/biology2020719 PMC3960892

[mbt214467-bib-0060] Gerday, C. , Aittaleb, M. , Bentahir, M. , Chessa, J.P. , Claverie, P. , Collins, T. et al. (2000) Cold‐adapted enzymes: from fundamentals to biotechnology. Trends in Biotechnology, 18(3), 103–107. 10675897 10.1016/s0167-7799(99)01413-4

[mbt214467-bib-0061] Ghosh, M. , Pulicherla, K.K. , Rekha, V.P. , Raja, P.K. & Sambasiva Rao, K.R.S. (2012) Cold active beta‐galactosidase from *Thalassospira* sp. 3SC‐21 to use in milk lactose hydrolysis: a novel source from deep waters of bay‐of‐Bengal. World Journal of Microbiology and Biotechnology, 28, 2859–2869. 22806727 10.1007/s11274-012-1097-z

[mbt214467-bib-0062] Glodowsky, A.P. , Ruberto, L.A. , Martorell, M.M. , Mac Cormack, W.P. & Levin, G.J. (2020) Cold active transglutaminase from antarctic *Penicillium chrysogenum*: partial purification, characterization and potential application in food technology. Biocatalysis and Agricultural Biotechnology, 29, 101807.

[mbt214467-bib-0063] Goodell, B. , Winandy, J.E. & Morrell, J.J. (2020) Fungal degradation of wood: emerging data, new insights and changing perceptions. Coatings, 10(12), 1210.

[mbt214467-bib-0064] Gounot, A.M. (1991) Bacterial life at low temperature: physiological aspects and biotechnological implications. The Journal of Applied Bacteriology, 71, 386–397. 1761432 10.1111/j.1365-2672.1991.tb03806.x

[mbt214467-bib-0065] Grzonka, Z. , Kasprzykowski, F. & Wiczk, W. (2007) Cysteine proteases. In: Polaina, J. & MacCabe, A.P. (Eds.) Industrial enzymes. Dordrecht: Springer.

[mbt214467-bib-0066] Guduk, E. , Yasar, G. , Guven Gulhan, U. & Aktaş, F. (2019) Isolation, purification and characterization of new cold active subtilisin‐like protease from *Bacillus* sp. strain EL‐GU1. Düzce Üniversitesi Bilim Ve Teknoloji Dergisi, 7, 2057–2073.

[mbt214467-bib-0067] Guo, C. , Zheng, R. , Cai, R. , Sun, C. & Wu, S. (2021) Characterization of two unique cold‐active lipases derived from a novel deep‐sea cold seep bacterium. Microorganisms, 9, 802. 33920298 10.3390/microorganisms9040802 PMC8069351

[mbt214467-bib-0069] Gupta, R. , Saini, V.K. , Bhatt, R.P. , Thapliyal, B.P. & Naithani, S. (2013) Influence of mechanical operation on the biodelignification of *Eucalyptus tereticornis* by *Trametes versicolor* . Cellulose Chemistry and Technology, 47, 759–764.

[mbt214467-bib-0070] Gupta, S.K. , Kataki, S. , Chatterjee, S. , Prasad, R.K. , Datta, S. , Vairale, M.G. et al. (2020) Cold adaptation in bacteria with a special focus on cellulase production and its potential application. Journal of Cleaner Production, 258, 120351.

[mbt214467-bib-0071] Gurung, N. , Ray, S. , Bose, S. & Rai, V. (2013) A broader view: microbial enzymes and their relevance in industries, medicine, and beyond. BioMed Research International, 3, 329121. 10.1155/2013/329121 PMC3784079 24106701

[mbt214467-bib-0072] Haile, S. & Ayele, A. (2022) Pectinase from microorganisms and its industrial applications. The Scientific World Journal, 2022, 1–15. Available from: 10.1155/2022/1881305 PMC8933074 35311220

[mbt214467-bib-0073] Hamdan, A. (2018) Psychrophiles: ecological significance and potential industrial application. South African Journal of Science, 114(5–6), 1–6.

[mbt214467-bib-0074] Hamid, B. (2021) Potent biotechnological applications of Psychrozymes. In: Microbiomes and the global climate change. Singapore: Springer, pp. 349–364.

[mbt214467-bib-0075] Hamid, B. , Bashir, Z. , Yatoo, A.M. , Mohiddin, F. , Majeed, N. , Bansal, M. et al. (2022) Cold‐active enzymes and their potential industrial applications—a review. Molecules, 27(18), 5885. 36144621 10.3390/molecules27185885 PMC9501442

[mbt214467-bib-0079] Hassan, S.W.M. , Abd El Latif, H.H. & Ali, S.M. (2018) Production of cold‐active lipase by free and immobilized marine *Bacillus cereus* HSS: application in wastewater treatment. Frontiers in Microbiology, 9, 2377. 30405541 10.3389/fmicb.2018.02377 PMC6205956

[mbt214467-bib-0080] Hildebrandt, P. , Wanarska, M. & Kur, J. (2009) A new cold‐adapted β‐D‐galactosidase from the Antarctic *Arthrobacter* sp. 32c – gene cloning, overexpression, purification and properties. BMC Microbiology, 9, 151. 19631003 10.1186/1471-2180-9-151 PMC2723119

[mbt214467-bib-0081] Hlalukana, N. , Magengelele, M. , Malgas, S. & Pletschke, B.I. (2021) Enzymatic conversion of mannan‐rich plant waste biomass into prebiotic mannooligosaccharides. Foods, 10(9), 2010. 34574120 10.3390/foods10092010 PMC8468410

[mbt214467-bib-3000] Hmidet, N. , Ali, N. , Haddar, A. , Kanoun, S. , Alya, S. & Nasri, M. (2009) Alkaline proteases and thermostable α‐amylase co‐produced by *Bacillus licheniformis* NH1: characterization and potential application as detergent additive. Biochemical Engineering Journal, 47, 71–79.

[mbt214467-bib-0082] Hosokawa, R. , Nagai, M. , Kondo, H. , Teragaki, J. & Okuyama, H. (2011) Application of autochthonous bioaugmentation in cold regions of Japan. In: Contaminated soils: environmental impact, disposal and treatment, pp. 463–472. New York, USA: Nova Science Publisher.

[mbt214467-bib-0084] Islam, M.N. , Karim, M.R. & Malinen, R.O. (2008) Beneficial effects of fungal treatment before pulping and bleaching of *Acacia mangium* and *Eucalyptus camaldulensis* . Turkish Journal of Agriculture and Forestry, 32, 331–338.

[mbt214467-bib-0085] Jaeger, K.E. & Eggert, T. (2002) Lipases for biotechnology. Current Opinion in Biotechnology, 13(4), 390–397. 12323363 10.1016/s0958-1669(02)00341-5

[mbt214467-bib-0086] Jeon, J.H. , Kim, J.‐T. , Kim, Y.J. , Kim, H.K. , Lee, H.S. , Kang, S.G. et al. (2009) Cloning and characterization of a new cold‐active lipase from a deep‐sea sediment metagenome. Applied Microbiology and Biotechnology, 81, 865–874. 18773201 10.1007/s00253-008-1656-2

[mbt214467-bib-0087] Jin, M. , Gai, Y. , Guo, X. , Hou, Y. & Zeng, R. (2019) Properties and applications of extremozymes from deep‐sea extremophilic microorganisms: a mini review. Marine Drugs, 17(12), 656. 31766541 10.3390/md17120656 PMC6950199

[mbt214467-bib-0089] Joseph, B. & Ramteke, P.W. (2013) Extracellular solvent stable cold‐active lipase from psychrotrophic *Bacillus sphaericus* MTCC 7526: partial purification and characterization. Annales de Microbiologie, 63, 363–370.

[mbt214467-bib-0090] Joseph, B. , Ramteke, P.W. & Thomas, G. (2008) Cold active microbial lipases: some hot issues and recent developments. Biotechnology Advances, 26, 457–470. 18571355 10.1016/j.biotechadv.2008.05.003

[mbt214467-bib-4000] Joseph, B. , Shrivastava, N. & Ramteke, P.W. (2012) Extracellular cold‐active lipase of *Microbacterium luteolum* isolated from Gangotri glacier, western Himalaya: isolation, partial purification and characterization. Journal of Genetic Engineering and Biotechnology, 10(1), 137–144.

[mbt214467-bib-0091] Joshi, S. & Satyanarayana, T. (2013) Biotechnology of cold‐active proteases. Biology, 2(2), 755–783. 24832807 10.3390/biology2020755 PMC3960895

[mbt214467-bib-5000] Karmakar, M. & Ray, R.R. (2011) Current trends in research and application of microbial cellulases. Research Journal of Microbiology, 6, 41–53.

[mbt214467-bib-0092] Kashyap, D. , Vohra, P. , Chopra, S. & Tewari, R. (2001) Applications of pectinases in the commercial sector: a review. Bioresource Technology, 77, 215–227. 11272008 10.1016/s0960-8524(00)00118-8

[mbt214467-bib-0094] Kavitha, M. (2016) Cold active lipases – an update. Frontiers in Life Science, 9(3), 226–238. Available from: 10.1080/21553769.2016.1209134

[mbt214467-bib-0095] Kim, H.J. , Park, S. , Lee, J.M. , Park, S. , Jung, W. , Kang, J.S. et al. (2008) *Moritella dasanensis* sp. nov., a psychrophilic bacterium isolated from the Arctic Ocean. International Journal of Systematic and Evolutionary Microbiology, 58(4), 817–820. 18398175 10.1099/ijs.0.65501-0

[mbt214467-bib-0096] Knoblauch, C. , Jørgensen, B.B. & Harder, J. (1999) Community size and metabolic rates of psychrophilic sulfate‐reducing bacteria in Arctic marine sediments. Applied and Environmental Microbiology, 65(9), 4230–4233. 10473441 10.1128/aem.65.9.4230-4233.1999 PMC99766

[mbt214467-bib-0097] Koutsioulis, D. , Wang, E. , Tzanodaskalaki, M. , Nikiforaki, D. , Deli, A. , Feller, G. et al. (2008) Directed evolution on the cold adapted properties of TAB5 alkaline phosphatase. Protein Engineering, Design & Selection, 21, 319–327. 10.1093/protein/gzn009 18411226

[mbt214467-bib-0098] Kuddus, M. (2015) Cold‐active microbial enzymes. Biochemistry & Physiology, 4, e132.

[mbt214467-bib-0099] Kuddus, M. (2018) Cold‐active enzymes in food biotechnology: an updated mini review. Journal of Applied Biology & Biotechnology, 6, 58–63.

[mbt214467-bib-0100] Kuddus, M. & Ahmad, I.Z. (2012) Cold‐active extracellular α‐amylase production from novel bacteria *Microbacterium foliorum* GA2 and *Bacillus cereus* GA6 isolated from Gangotri glacier, Western Himalaya. Journal, Genetic Engineering & Biotechnology, 10(1), 151–159.

[mbt214467-bib-6000] Kuddus, M. & Ramteke, P.W. (2008) A cold‐active extracellular metalloprotease from *Curtobacterium luteum* (MTCC7529): enzyme production and characterization. The Journal of General and Applied Microbiology, 54(6), 385–392. 19164881 10.2323/jgam.54.385

[mbt214467-bib-7000] Kuddus, M. & Ramteke, P.W. (2009) Cold‐active extracellular alkaline protease from an alkaliphilic *Stenotrophomonas maltophilia*: production of enzyme and its industrial applications. Canadian Journal of Microbiology, 55(11), 1294–1301. 19940938 10.1139/w09-089

[mbt214467-bib-0101] Kuddus, M. & Ramteke, P.W. (2011) Production optimization of an extracellular cold‐active alkaline protease from *Stenotrophomonas maltophilia* MTCC 7528 and its application in detergent industry. African Journal of Microbiology Research, 5(7), 809–816.

[mbt214467-bib-0102] Kuddus, M. , Roohi, A.J. & Ramteke, P.W. (2012) Structural adaptation and biocatalytic prospective of microbial cold‐active α‐amylase. African Journal of Microbiology Research, 6, 206–213.

[mbt214467-bib-0103] Kuddus, M. , Roohi, A.J.M. & Ramteke, P.W. (2011) An overview of cold‐active microbial α‐amylase: adaptation strategies and biotechnological potentials. Biotech, 10, 246–258.

[mbt214467-bib-0104] Kuhn, E. , Bellicanta, G.S. & Pellizari, V.H. (2009) New alk genes detected in Antarctic marine sediments. Environmental Microbiology, 11, 669–673. 19207566 10.1111/j.1462-2920.2008.01843.x

[mbt214467-bib-0105] Kumar, A. , Mukhia, S. & Kumar, R. (2021) Industrial applications of cold‐adapted enzymes: challenges, innovations and future perspective. 3 Biotech, 11(10), 426. 10.1007/s13205-021-02929-y PMC8421504 34567931

[mbt214467-bib-0106] Kumar, A. , Gautam, A. & Dutt, D. (2020) Bio‐pulping: an energy saving and environment‐friendly approach. Physical Sciences Reviews, 5(10), 20190043.

[mbt214467-bib-0107] Kumar, N.V. & Rani, M.E. (2019) Microbial enzymes in paper and pulp industries for bioleaching application. MedDocs eBooks. Nevada, USA: MedDocs Publisher LLC.

[mbt214467-bib-0108] Kumari, M. , Padhi, S. , Sharma, S. , Phukon, L.C. , Singh, S.P. & Rai, A.K. (2021) Biotechnological potential of psychrophilic microorganisms as the source of cold‐active enzymes in food processing applications. 3 Biotech, 11, 1–18. 10.1007/s13205-021-03008-y PMC8557216 34790503

[mbt214467-bib-0109] Kumari, S. , Kumar, A. & Kumar, R. (2023) A cold‐active cellulase produced from *Exiguobacterium sibiricum* K1 for the valorization of agro‐residual resources. Biomass Conversion and Biorefinery, 13, 14777–14787.

[mbt214467-bib-0112] Lanes, O. , Leiros, I. , Smalas, A.O. & Willassen, N.P. (2002) Identification, cloning, and expression of uracil‐DNA glycosylase from Atlantic cod (*Gadus morhua*): characterization and homology modeling of the cold‐active catalytic domain. Extremophiles, 6, 73–86. 11878565 10.1007/s007920100225

[mbt214467-bib-0113] Le, L.T.H.L. , Yoo, W. , Jeon, S. , Lee, C. , Kim, K.K. , Lee, J.H. et al. (2020) Biodiesel and flavor compound production using a novel promiscuous cold‐adapted SGNH‐type lipase (HaSGNH1) from the psychrophilic bacterium *Halocynthiibacter arcticus* . Biotechnology for Biofuels, 13, 1–13. 32190120 10.1186/s13068-020-01696-x PMC7074997

[mbt214467-bib-0114] Lee, C.W. , Park, S.H. , Jeong, C.S. , Cha, S.S. , Park, H. & Lee, J.H. (2019) Structural basis of small RNA hydrolysis by oligoribonuclease (CpsORN) from *Colwellia psychrerythraea* strain 34H. Scientific Reports, 9, 2649. 30804410 10.1038/s41598-019-39641-0 PMC6390093

[mbt214467-bib-0115] Lee, D.H. , Choi, S.L. , Rha, E. , Kim, S. , Yeom, S.J. , Moon, J.H. et al. (2015) A novel psychrophilic alkaline phosphatase from the metagenome of tidal flat sediments. BMC Biotechnology, 15, 1. 25636680 10.1186/s12896-015-0115-2 PMC4335783

[mbt214467-bib-0116] Lee, M.S. , Kim, G.A. , Seo, M.S. , Lee, J.H. & Kwon, S.T. (2009) Characterization of heat‐labile uracil‐DNA glycosylase from *Psychrobacter* sp. HJ147 and its application to the polymerase chain reaction. Biotechnology and Applied Biochemistry, 52(Pt 2), 167–175. 18412541 10.1042/BA20080028

[mbt214467-bib-0117] Lee, S.H. , Son, H.F. & Kim, K.J. (2019) Structural insights into the inhibition properties of archaeon citrate synthase from *Metallosphaera sedula* . PLoS One, 14, e0212807. 30794680 10.1371/journal.pone.0212807 PMC6386500

[mbt214467-bib-0118] Lehr, M. , Miltner, M. & Friedl, A. (2021) Removal of wood extractives as pulp (pre‐)treatment: a technological review. SN Applied Sciences, 3, 886.

[mbt214467-bib-0119] Li, X. , She, Y. , Sun, B. , Song, H. , Zhu, Y. , Lv, Y. et al. (2010) Purification and characterization of a cellulase‐free, thermostable xylanase from *Streptomyces rameus* L2001 and its biobleaching effect on wheat straw pulp. Biochemical Engineering Journal, 52(1), 71–78.

[mbt214467-bib-0120] Li, Y. , Wang, Z. , Zhou, Y. , Zhu, G. & Lin, L. (2019) Enzymatic identification and functional sites study of a novel cold‐active cellulase (MkCel5) from *Microbacterium kitamiensea* . Biotechnology & Biotechnological Equipment, 33(1), 739–747.

[mbt214467-bib-0123] Maciejewska, N. , Walkusz, R. , Olszewski, M. & Szymańska, A. (2019) New nuclease from extremely psychrophilic microorganism *Psychromonas ingrahamii* 37: identification and characterization. Molecular Biotechnology, 61, 122–133. 30539415 10.1007/s12033-018-0142-z

[mbt214467-bib-0124] Mageswari, A. , Subramanian, P. , Chandrasekaran, S. , Karthikeyan, S. & Gothandam, K.M. (2017) Systematic functional analysis and application of a cold‐active serine protease from a novel *Chryseobacterium* sp. Food Chemistry, 217, 18–27. 27664603 10.1016/j.foodchem.2016.08.064

[mbt214467-bib-0125] Mahto, K.U. & Das, S. (2022) Bacterial biofilm and extracellular polymeric substances in the moving bed biofilm reactor for wastewater treatment: a review. Bioresource Technology, 345, 126476. 34864174 10.1016/j.biortech.2021.126476

[mbt214467-bib-0126] Maijala, P. , Kleen, M. , Westin, C. , Poppius‐Levlin, K. , Herranen, K. , Lehto, J.H. et al. (2008) Biomechanical pulping of softwood with enzymes and white‐rot fungus *Physisporinus rivulosus* . Enzyme and Microbial Technology, 43, 169–177.

[mbt214467-bib-0128] Mangiagalli, M. & Lotti, M. (2021) Cold‐active β‐galactosidases: insight into cold adaptation mechanisms and biotechnological exploitation. Marine Drugs, 19(1), 43. 33477853 10.3390/md19010043 PMC7832830

[mbt214467-bib-0129] Margesin, R. (2009) Effect of temperature on growth parameters of psychrophilic bacteria and yeasts. Extremophiles, 13, 257–262. 19057843 10.1007/s00792-008-0213-3

[mbt214467-bib-0130] Margesin, R. & Collins, T. (2019) Microbial ecology of the cryosphere (glacial and permafrost habitats): current knowledge. Applied Microbiology and Biotechnology, 103, 2537–2549. 30719551 10.1007/s00253-019-09631-3 PMC6443599

[mbt214467-bib-0131] Margesin, R. , Feller, G. , Gerday, C. & Russell, N.J. (2002) Cold‐adapted microorganisms: adaptation strategies and biotechnological potential. In: Bitton, G. (Ed.) The encyclopedia of environmental microbiology, Vol. 2. New York: Wiley, pp. 871–888.

[mbt214467-bib-0134] Martínez Álvarez, L.M. , Ruberto, L. , Lo Balbo, A. & Mac Cormack, W.P. (2017) Bioremediation of hydrocarbon‐contaminated soils in cold regions: development of a pre‐optimized biostimulation biopile‐scale field assay in Antarctica. Science of the Total Environment, 590‐591, 194–203. 10.1016/j.scitotenv.2017.02.204 28262358

[mbt214467-bib-0135] Marx, J.C. , Collins, T. , D'Amico, S. , Feller, G. & Gerday, C. (2007) Cold‐adapted enzymes from marine Antarctic microorganisms. Marine Biotechnology, 9, 293–304. 17195087 10.1007/s10126-006-6103-8

[mbt214467-bib-0136] Mendonça, R. , Jara, J.F. , González, V. , Elissetche, J.P. & Freer, J. (2008) Evaluation of the white‐rot fungi *Ganoderma australe* and *Ceriporiopsis subvermispora* in biotechnological applications. Journal of Industrial Microbiology & Biotechnology, 35, 1323–1330. 18712558 10.1007/s10295-008-0414-x

[mbt214467-bib-0137] Mesbah, N.M. (2022) Industrial biotechnology based on enzymes from extreme environments. Frontiers in Bioengineering and Biotechnology, 10. Available from: 10.3389/fbioe.2022.870083 PMC9036996 35480975

[mbt214467-bib-0138] Miri, S. , Davoodi, S.M. , Robert, T. , Brar, S.K. , Martel, R. & Rouissi, T. (2022) Enzymatic biodegradation of highly p‐xylene contaminated soil using cold‐active enzymes: a soil column study. Journal of Hazardous Materials, 423(Pt A), 127099. 34523486 10.1016/j.jhazmat.2021.127099

[mbt214467-bib-0139] Miri, S. , Perez, J.A.E. , Brar, S.K. , Rouissi, T. & Martel, R. (2021) Sustainable production and co‐immobilization of cold‐active enzymes from *Pseudomonas* sp. for BTEX biodegradation. Environmental Pollution (Barking, Essex: 1987), 285, 117678. 34380234 10.1016/j.envpol.2021.117678

[mbt214467-bib-0140] Moharram, A.M. , Zohri, A.N.A. , Hesham, A.E.L. , Hesham, A.E.L. , Abdel‐Raheam, H.E.F. , Al‐Ameen Maher, M. et al. (2022) Production of cold‐active pectinases by three novel *Cladosporium* species isolated from Egypt and application of the most active enzyme. Scientific Reports, 12, 15599. 36114347 10.1038/s41598-022-19807-z PMC9481535

[mbt214467-bib-0141] Moyer, C.L. & Morita, R.Y. (2007) Psychrophiles and psychrotrophs. In: Encyclopedia of life sciences. New Jersey, USA: Wiley Online Library. Available from: 10.1002/9780470015902.a0000402.pub2

[mbt214467-bib-0142] Mukhia, S. , Kumar, A. & Kumar, R. (2021) Generation of antioxidant peptides from soy protein isolate through psychrotrophic *Chryseobacterium* sp. derived alkaline broad temperature active protease. LWT – Food Science and Technology, 143, 111–152.

[mbt214467-bib-0143] Mykytczuk, N.C. , Foote, S.J. , Omelon, C.R. , Southam, G. , Greer, C.W. & Whyte, L.G. (2013) Bacterial growth at −15°C; molecular insights from the permafrost bacterium *Planococcus halocryophilus* Or1. The ISME Journal, 7, 1211–1226. 23389107 10.1038/ismej.2013.8 PMC3660685

[mbt214467-bib-0144] Nandanwar, S.K. , Borkar, S.B. , Lee, J.H. & Kim, H.J. (2020) Taking advantage of promiscuity of cold‐active enzymes. Applied Sciences, 2020(10), 1–18.

[mbt214467-bib-0145] Neklyudov, A.D. , Ivankin, A.N. & Berdutina, A.V. (2000) Properties and uses of protein hydrolysates (review). Applied Biochemistry and Microbiology, 36, 452–459.

[mbt214467-bib-0146] Nimkande, V.D. & Bafana, A. (2022) A review on the utility of microbial lipases in wastewater treatment. Journal of Water Process Engineering, 46, 102591.

[mbt214467-bib-8000] Niyonzima, F.N. (2019) Detergent‐compatible bacterial cellulases. Journal of Basic Microbiology, 59(2), 134–147. 30421443 10.1002/jobm.201800436

[mbt214467-bib-9000] Niyonzima, F.N. & More, S.S. (2014) Concomitant production of detergent compatible enzymes by *Bacillus flexus* XJU‐1. Brazilian Journal of Microbiology, 45(3), 903–910. 25477924 10.1590/s1517-83822014000300020 PMC4204975

[mbt214467-bib-0147] Núñez‐Montero, K. , Salazar, R. , Santos, A. , Gómez‐Espinoza, O. , Farah, S. , Troncoso, C. et al. (2021) Antarctic *Rahnella inusitata*: a producer of cold‐stable β‐galactosidase enzymes. International Journal of Molecular Sciences, 22(8), 4144. 33923711 10.3390/ijms22084144 PMC8074230

[mbt214467-bib-1010] Pandey, A. , Benjamin, S. , Soccol, C.R. , Nigam, P. , Krieger, N. & Soccol, V.T. (1999) The realm of microbial lipases in biotechnology. Biotechnology and Applied Biochemistry, 29(2), 119–131. 10075908

[mbt214467-bib-0148] Pandey, L.K. , Kumar, A. , Dutt, D. & Singh, S.P. (2022) Influence of mechanical operation on the biodelignification of *Leucaena leucocephala* by xylanase treatment. 3 Biotech, 12(1), 1–13. 34956813 10.1007/s13205-021-03024-y PMC8677879

[mbt214467-bib-0149] Park, C. & Park, W. (2018) Survival and energy producing strategies of alkane degraders under extreme conditions and their biotechnological potential. Frontiers in Microbiology, 9, 1081. 29910779 10.3389/fmicb.2018.01081 PMC5992423

[mbt214467-bib-0150] Park, H.J. , Lee, C.W. , Kim, D. , Do, H. , Han, S.J. , Kim, J.E. et al. (2018) Crystal structure of a cold‐active protease (Pro21717) from the psychrophilic bacterium, *Pseudoalteromonas arctica* PAMC 21717, at 1.4 Å resolution: structural adaptations to cold and functional analysis of a laundry detergent enzyme. PLoS One, 13(2), e0191740. 29466378 10.1371/journal.pone.0191740 PMC5821440

[mbt214467-bib-0151] Parmar, T.S. , Ahirwar, R. & Sahay, S. (2023) Isolation, purification and characterization of carboxymethyl cellulase (CMCase) from psychrotolerant yeast *Rhodotorula mucilaginosa* BPT1. Materials Today Proceedings, 72, 2768–2772.

[mbt214467-bib-0152] Parra, L.P. , Reyes, F. , Acevedo, J.P. , Salazar, O. , Andrews, B.A. & Asenjo, J.A. (2008) Cloning and fusion expression of a cold‐active lipase from marine Antarctic origin. Enzyme and Microbial Technology, 42, 371–377.

[mbt214467-bib-0153] Patel, A.B. , Mahala, K. , Jain, K. & Madamwar, D. (2018) Development of mixed bacterial cultures DAK11 capable for degrading mixture of polycyclic aromatic hydrocarbons (PAHs). Bioresource Technology, 253, 288–296. 29353758 10.1016/j.biortech.2018.01.049

[mbt214467-bib-0154] Peng, Y. , He, W. , Li, Y. , Liu, L. , Deng, B. , Yan, G. et al. (2022) Degradation of CP4‐EPSPS with a psychrophilic bacterium *Stenotrophomonas maltophilia* 780. Biomolecules, 12(2), 318. 35204818 10.3390/biom12020318 PMC8869762

[mbt214467-bib-0155] Perfumo, A. , Freiherr von Sass, G.J. , Nordmann, E.‐L. , Budisa, N. & Wagner, D. (2020) Discovery and characterization of a new cold‐active protease from an extremophilic bacterium via comparative genome analysis and in vitro expression. Frontiers in Microbiology, 11, 881. 32528424 10.3389/fmicb.2020.00881 PMC7247812

[mbt214467-bib-0156] Poveda, G. , Gil‐Durán, C. , Vaca, I. , Levicán, G. & Chávez, R. (2018) Cold‐active pectinolytic activity produced by filamentous fungi associated with Antarctic marine sponges. Biological Research, 51, 28. 30149803 10.1186/s40659-018-0177-4 PMC6109986

[mbt214467-bib-0157] Pulicherla, K. , Ghosh, M. , Kumar, P. & Rao, K. (2011) Psychrozymes—the next generation industrial enzymes. Journal of Marine Science: Research & Development, 1, 2.

[mbt214467-bib-0158] Pulicherla, K.K. , Kumar, P.S. , Manideep, K. , Rekha, V.P. , Ghosh, M. & Sambasiva Rao, K.R. (2013) Statistical approach for the enhanced production of cold‐active beta‐galactosidase from *Thalassospira frigidphilosprofundus*: a novel marine psychrophile from deep waters of bay of Bengal. Preparative Biochemistry & Biotechnology, 43, 766–780. 23876137 10.1080/10826068.2013.773341

[mbt214467-bib-0159] Rahman, M.M. , Mazumder, N.U.S. , Ferdousi, U.S. , Shahid, M.A. & Hoque, M.B. (2023) Application of biochemical in textile. In: Advanced technology in textiles: fibre to apparel. Singapore: Springer Nature Singapore, pp. 301–321.

[mbt214467-bib-0161] Reid, I. & Ricard, M. (2002) Pectinase in papermaking: solving retention problems in mechanical pulp bleached with hydrogen peroxide. Enzyme and Microbial Technology, 26, 115–123. 10.1016/s0141-0229(99)00131-3 10689066

[mbt214467-bib-0162] Rittié, L. & Perbal, B. (2008) Enzymes used in molecular biology: a useful guide. Journal of Cell Communication and Signaling, 2(1–2), 25–45. 18766469 10.1007/s12079-008-0026-2 PMC2570007

[mbt214467-bib-0163] Rojas‐Contreras, J. , de la Rosa, A.P. & De Leon‐Rodriguez, A. (2015) Expression and characterization of a recombinant psychrophilic Cu/Zn superoxide dismutase from *Deschampsia Antarctica* E. Desv. [Poaceae]. Applied Biochemistry and Biotechnology, 175, 3287–3296. 25638267 10.1007/s12010-015-1496-3

[mbt214467-bib-0164] Rota, E. , Bergami, E. , Corsi, I. & Bargagli, R. (2022) Macro‐ and microplastics in the Antarctic environment: ongoing assessment and perspectives. Environments, 9, 93.

[mbt214467-bib-0165] Rutkiewicz, M. , Bujacz, A. & Bujacz, G. (2019) Structural features of cold‐adapted dimeric GH2 β‐D‐galactosidase from *Arthrobacter* sp. 32cB. Biochimica et Biophysica Acta (BBA)‐Proteins and Proteomics, 1867(9), 776–786. 31195142 10.1016/j.bbapap.2019.06.001

[mbt214467-bib-0167] Sahay, S. , Hamid, B. , Singh, P. , Ranjan, K. , Chauhan, D. , Rana, R.S. et al. (2013) Evaluation of pectinolytic activities for oenological uses from psychrotrophic yeasts. Letters in Applied Microbiology, 57(2), 115–121. 23574042 10.1111/lam.12081

[mbt214467-bib-0169] Sánchez, L.A. , Gómez, F.F. & Delgado, O.D. (2009) Cold‐adapted microorganisms as a source of new antimicrobials. Extremophiles, 13(1), 111–120. 19015813 10.1007/s00792-008-0203-5

[mbt214467-bib-0170] Santiago, M. , Ramírez‐Sarmiento, C.A. , Zamora, R.A. & Parra, L.P. (2016) Discovery, molecular mechanisms, and industrial applications of cold‐active enzymes. Frontiers in Microbiology, 7, 1408. 27667987 10.3389/fmicb.2016.01408 PMC5016527

[mbt214467-bib-0171] Sarmiento, F. , Peralta, R. & Blamey, J.M. (2015) Cold and hot extremozymes: industrial relevance and current trends. Frontiers in Bioengineering and Biotechnology, 3, 148. 26539430 10.3389/fbioe.2015.00148 PMC4611823

[mbt214467-bib-0172] Schäfer, T. , Borchert, T.W. , Nielsen, V.S. , Skagerlind, P. , Gibson, K. , Wenger, K. et al. (2006) Industrial enzymes. In: Ulber, R. & Sell, D. (Eds.) White biotechnology. Advances in biochemical engineering/biotechnology, Vol. 105. Berlin, Heidelberg: Springer. 10.1007/10_2006_039 17408082

[mbt214467-bib-0173] Schmidt‐Nielsen, S. (1902) Ueber einige psychrophile Mikroorganismen und ihr Vorkommen. Centralblatt für Bacteriologie und Parasitenkunde Abt II, 9, 145–147.

[mbt214467-bib-0174] Schormann, N. , Ricciardi, R. & Chattopadhyay, D. (2014) Uracil‐DNA glycosylases—structural and functional perspectives on an essential family of DNA repair enzymes. Protein Science: A Publication of the Protein Society, 23(12), 1667–1685. 25252105 10.1002/pro.2554 PMC4253808

[mbt214467-bib-0177] Semenova, M. et al. (2006) Use of a preparation from fungal pectin lyase in the food industry. Applied Biochemistry and Microbiology, 42, 598–602. 17168297

[mbt214467-bib-0178] Sharma, U. , Pal, D. & Prasad, R. (2014) Alkaline phosphatase: an overview. Indian Journal of Clinical Biochemistry, 29(3), 269–278. 24966474 10.1007/s12291-013-0408-y PMC4062654

[mbt214467-bib-0179] Shrinivas, D. , Savitha, G. , Raviranjan, K. & Naik, G.R. (2010) A highly thermostable alkaline cellulase‐free xylanase from thermoalkalophilic *Bacillus* sp. JB 99 suitable for paper and pulp industry: purification and characterization. Applied Biochemistry and Biotechnology, 162(7), 2049–2057. 20467831 10.1007/s12010-010-8980-6

[mbt214467-bib-0180] Shukla, T.P. & Wierzbicki, L.E. (1975) β‐Galactosidase technology: a solution to the lactose problem. Food Science & Nutrition, 25, 325–356.

[mbt214467-bib-0181] Simpanen, S. , Dahl, M. , Gerlach, M. , Mikkonen, A. , Malk, V. , Mikola, J. et al. (2016) Biostimulation proved to be the most efficient method in the comparison of in situ soil remediation treatments after a simulated oil spill accident. Environmental Science and Pollution Research International, 23(24), 25024–25038. 27677992 10.1007/s11356-016-7606-0 PMC5124059

[mbt214467-bib-0182] Singh, R. , Mittal, A. , Kumar, M. & Mehta, P. (2016) Amylases: a note on current applications. International Research Journal of Biological Sciences, 5, 27–32.

[mbt214467-bib-0183] Singhal, P. , Nigam, K.V. & Vidyarthi, S.A. (2012) Studies on production, characterization and applications of microbial alkaline protease. International Journal of Advanced Biotechnology and Research, 3, 653–669.

[mbt214467-bib-0184] Smirnova, M. , Losada, C.B. , Akulava, V. , Zimmermann, B. , Kohler, A. , Miamin, U. et al. (2023) New cold‐adapted bacteria for efficient hydrolysis of feather waste at low temperature. Bioresource Technology Reports, 23, 101530. Available from: 10.1016/j.biteb.2023.101530

[mbt214467-bib-0185] Soares, M.M.C.N. (2001) Pectinolytic enzyme production by *Bacillus* sp. and their potential application on juice extraction. World Journal of Microbiology and Biotechnology, 17, 79–82.

[mbt214467-bib-0187] Stallwood, B. , Shears, J. , Williams, P.A. & Hughes, K.A. (2005) Low temperature bioremediation of oil‐contaminated soil using biostimulation and bioaugmentation with a pseudomonas sp. from maritime Antarctica. Journal of Applied Microbiology, 99(4), 794–802. 16162230 10.1111/j.1365-2672.2005.02678.x

[mbt214467-bib-0189] Struvay, C. & Feller, G. (2012) Optimization to low temperature activity in psychrophilic enzymes. International Journal of Molecular Sciences, 13(9), 11643–11665. 23109875 10.3390/ijms130911643 PMC3472767

[mbt214467-bib-0190] Suhaib, M.M. , Farooq, H.A. , Sanjay, S. & Kirti, J. (2018) Industrial applications and production of cold active xylanase: a review. International Journal of Research and Analytical Reviews, 5(3), 1215–1224.

[mbt214467-bib-0191] Tan, S. , Owusu, A.R.K. & Knapp, J. (1996) Low temperature organic phase biocatalysis using cold adapted lipase from psychrotrophic *Pseudomonas* P38. Food Chemistry, 57, 415–418.

[mbt214467-bib-0193] Tetzner, R. (2009) Prevention of PCR cross‐contamination by UNG treatment of bisulfite‐treated DNA. Methods in Molecular Biology (Clifton, N.J.), 507, 357–370. 10.1007/978-1-59745-522-0_26 18987827

[mbt214467-bib-0195] Tratsiak, K. , Prudnikova, T. , Drienovska, I. , Damborsky, J. , Brynda, J. , Pachl, P. et al. (2019) Crystal structure of the cold‐adapted haloalkane dehalogenase DpcA from *Psychrobacter cryohalolentis* K5. Acta Crystallographica. Section F, 75, 324–331. 10.1107/S2053230X19002796 PMC6497103 31045561

[mbt214467-bib-0196] Tribelli, P.M. , Pezzoni, M. , Brito, M.G. , Montesinos, N.V. , Costa, C.S. & López, N.I. (2020) Response to lethal UVA radiation in the Antarctic bacterium *Pseudomonas extremaustralis*: polyhydroxybutyrate and cold adaptation as protective factors. Extremophiles, 24, 265–275. 31828543 10.1007/s00792-019-01152-1

[mbt214467-bib-0197] Valls, C. , Vidal, T. & Roncero, M.B. (2010) The role of xylanases and laccases on hexenuronic acid and lignin removal. Process Biochemistry, 45(3), 425–430.

[mbt214467-bib-0198] Vester, J.K. , Glaring, M.A. & Stougaard, P. (2015) Improved cultivation and metagenomics as new tools for bioprospecting in cold environments. Extremophiles, 19, 17–29. 25399309 10.1007/s00792-014-0704-3 PMC4272415

[mbt214467-bib-0199] Wackett, L.P. (2019) Microbial industrial enzymes: an annotated selection of world wide web sites relevant to the topics in microbial biotechnology. Microbial Biotechnology, 12(5), 1090–1091. 31380613 10.1111/1751-7915.13469 PMC6681399

[mbt214467-bib-0200] Walia, A. , Guleria, S. , Mehta, P. , Chauhan, A. & Parkash, J. (2017) Microbial xylanases and their industrial application in pulp and paper biobleaching: a review. 3 Biotech, 7(1), 1–12. 10.1007/s13205-016-0584-6 PMC5385172 28391477

[mbt214467-bib-0204] Wang, J. , Zhu, M. , Wang, P. & Chen, W. (2023) Biochemical properties of a cold‐active chitinase from marine *Trichoderma gamsii* R1 and its application to preparation of chitin oligosaccharides. Marine Drugs, 21, 332. 37367657 10.3390/md21060332 PMC10302006

[mbt214467-bib-0206] Wang, Q. , Hou, Y. , Ding, Y. & Yan, P. (2012) Purification and biochemical characterization of a cold‐active lipase from Antarctic Sea ice bacteria *Pseudoalteromonas* sp. NJ 70. Molecular Biology Reports, 39, 9233–9238. 22714922 10.1007/s11033-012-1796-4

[mbt214467-bib-0207] Wang, Y. , Hou, Y. , Nie, P. , Wang, Y. , Ren, X. , Wei, Q. et al. (2019) A novel cold‐adapted and salt‐tolerant RNase R from Antarctic sea‐ice bacterium *Psychrobacter* sp. Ant206. Molecules, 24, 2229. 31207974 10.3390/molecules24122229 PMC6630635

[mbt214467-bib-0208] Wei, S. , Liu, K. , Ji, X. , Wang, T. & Wang, R. (2021) Application of enzyme technology in biopulping and biobleaching. Cellulose, 28, 10099–10116.

[mbt214467-bib-0209] Worku, L.A. , Bachheti, A. , Bachheti, R.K. , Rodrigues Reis, C.E. & Chandel, A.K. (2023) Agricultural residues as raw materials for pulp and paper production: overview and applications on membrane fabrication. Membranes, 2023(13), 228. 10.3390/membranes13020228 PMC9959550 36837731

[mbt214467-bib-0210] Wu, Y. , Huang, S. , Liang, X. , Han, P. & Liu, Y. (2023) Characterization of a novel detergent‐resistant type I pullulanase from *Bacillus megaterium* Y103 and its application in laundry detergent. Preparative Biochemistry & Biotechnology, 53(6), 683–689. 36271878 10.1080/10826068.2022.2134890

[mbt214467-bib-0212] Yang, S.‐Z. , Jin, H.‐J. , Wei, Z. , He, R.‐X. , Ji, Y.‐J. , Li, X.‐M. et al. (2009) Bioremediation of oil spills in cold environments: a review. Pedosphere, 19(3), 371–381.

[mbt214467-bib-0213] Yang, W. (2011) Nucleases: diversity of structure, function and mechanism. Quarterly Reviews of Biophysics, 44(1), 1–93. 20854710 10.1017/S0033583510000181 PMC6320257

[mbt214467-bib-0214] Yang, Z. , Huang, Z. , Wu, Q. , Tang, X. & Huang, Z. (2023) Cold‐adapted proteases: an efficient and energy‐saving biocatalyst. International Journal of Molecular Sciences, 24, 8532. 37239878 10.3390/ijms24108532 PMC10218192

[mbt214467-bib-0215] Yao, C. , Sun, J. , Wang, W. , Zhuang, Z. , Liu, J. & Hao, J. (2019) A novel cold‐adapted β‐galactosidase from *Alteromonas* sp. ML117 cleaves milk lactose effectively at low temperature. Process Biochemistry, 82, 94–101.

[mbt214467-bib-0216] Yayanos, A.A. (1995) Microbiology to 10,500 meters in the deep sea. Annual Review of Microbiology, 49(1), 777–805. 10.1146/annurev.mi.49.100195.004021 8561479

[mbt214467-bib-0218] Yuan, X. , Zhang, X. , Chen, X. , Kong, D. , Liu, X. & Shen, S. (2018) Synergistic degradation of crude oil by indigenous bacterial consortium and exogenous fungus *Scedosporium boydii* . Bioresource Technology, 264, 190–197. 29803810 10.1016/j.biortech.2018.05.072

[mbt214467-bib-0219] Zakaria, M.R. , Hirata, S. , Fujimoto, S. & Hassan, M.A. (2015) Combined pretreatment with hot compressed water and wet disk milling opened up oil palm biomass structure resulting in enhanced enzymatic digestibility. Bioresource Technology, 193, 128–134. 26125612 10.1016/j.biortech.2015.06.074

[mbt214467-bib-0220] Zerva, A. , Simić, S. & Topakas, E. (2019) Applications of microbial laccases: patent review of the past decade (2009–2019). Catalysts, 9(12), 1023.

[mbt214467-bib-0221] Zhang, A. , Hou, Y. , Wang, Y. , Wang, Q. , Shan, X. & Liu, J. (2023) Highly efficient low‐temperature biodegradation of polyethylene microplastics by using cold‐active laccase cell‐surface display system. Bioresource Technology, 382, 129164. 37207695 10.1016/j.biortech.2023.129164

[mbt214467-bib-0222] Zhang, D.C. , Li, H.R. , Xin, Y.H. , Chi, Z.M. , Zhou, P.J. & Yu, Y. (2008) *Marinobacter psychrophilus* sp. nov., a psychrophilic bacterium isolated from the Arctic. International Journal of Systematic and Evolutionary Microbiology, 58(6), 1463–1466. 18523195 10.1099/ijs.0.65690-0

[mbt214467-bib-0223] Zhang, D.C. , Li, H.R. , Xin, Y.H. , Liu, H.C. , Chi, Z.M. , Zhou, P.J. et al. (2008) *Phaeobacter arcticus* sp. nov., a psychrophilic bacterium isolated from the Arctic. International Journal of Systematic and Evolutionary Microbiology, 58(6), 1384–1387. 18523182 10.1099/ijs.0.65708-0

[mbt214467-bib-0224] Zhang, D.C. , Liu, H.C. , Xin, Y.H. , Yu, Y. , Zhou, P.J. & Zhou, Y.G. (2008) *Salinibacterium xinjiangense* sp. nov., a psychrophilic bacterium isolated from the China No. 1 Glacier. International Journal of Systematic and Evolutionary Microbiology, 58(12), 2739–2742. 19060050 10.1099/ijs.0.65802-0

[mbt214467-bib-0228] Zhou, Y. , Chen, X. , Li, X. , Han, Y. , Wang, Y. , Yao, R. et al. (2019) Purification and characterization of a new cold‐adapted and thermo‐tolerant chitosanase from marine bacterium *Pseudoalteromonas* sp. SY39. Molecules, 24, 183. 30621320 10.3390/molecules24010183 PMC6337222

